# Effects of Using Websites on Physical Activity and Diet Quality for Adults Living With Chronic Health Conditions: Systematic Review and Meta-Analysis

**DOI:** 10.2196/49357

**Published:** 2023-10-19

**Authors:** Dina Pogrebnoy, Amy M Dennett, Dawn B Simpson, Lesley MacDonald-Wicks, Amanda J Patterson, Coralie English

**Affiliations:** 1 School of Health Sciences, University of Newcastle Newcastle Australia; 2 Department of Physiotherapy Western Health St Albans Australia; 3 Allied Health Clinical Research Office Eastern Health Melbourne Australia; 4 School of Allied Health, Human Services and Sport Latrobe University Melbourne Australia; 5 Heart and Stroke Program Hunter Medical Research Institute Newcastle Australia; 6 Food and Nutrition Program Hunter Medical Institute Newcastle Australia

**Keywords:** modifiable risk factors, secondary prevention, physical activity, diet quality, websites, chronic health, chronic illness

## Abstract

**Background:**

Adults with chronic health conditions need support to manage modifiable risk factors such as physical inactivity and poor diet. Disease-specific websites with health information on physical activity and diet quality may be effective in supporting adults in managing their chronic illnesses.

**Objective:**

The primary aim of this review was to determine whether using websites with health information can lead to improvements in physical activity levels or diet quality in adults with chronic health conditions.

**Methods:**

Randomized controlled trials evaluating the effectiveness of website use on levels of physical activity or diet quality in adults with chronic health conditions were included. MEDLINE, Embase, CINAHL, and the Physiotherapy Evidence Database were searched from the earliest available record until February 2023. Data for outcomes measuring physical activity levels; diet quality; and, where reported, self-efficacy and quality of life were independently extracted by 2 reviewers. The risk of bias was assessed using the Physiotherapy Evidence Database scale, and the overall certainty of evidence was assessed using the Grading of Recommendations Assessment, Development, and Evaluation approach. Where values were presented as the same unit of measure, postintervention scores were pooled for meta-analysis to yield an overall mean difference (MD). A standardized MD (SMD) was calculated for the pooled data in which different units for the same outcome were used. Individual trial data were described in cases where the data of trials could not be pooled.

**Results:**

A total of 29 trials (N=6418 participants) across 8 different disease groups with intervention periods ranging from 4 weeks to 12 months were included in the analysis. There was moderate-certainty evidence that using websites with health information increased levels of moderate to vigorous physical activity (MD=39 min/wk, 95% CI 18.60-58.47), quality of life (SMD=0.36, 95% CI 0.12-0.59), and self-efficacy (SMD=0.26, 95% CI 0.05-0.48) and high-certainty evidence for reduction in processed meat consumption (MD=1.1 portions/wk, 95% CI 0.70-1.58) when compared with usual care. No differences were detected in other measures of diet quality. There was no increased benefit for website users who were offered additional support.

**Conclusions:**

The use of websites for risk factor management has the potential to improve physical activity levels, quality of life, and self-efficacy as well as reduce processed meat consumption for adults living with chronic health conditions when compared with usual care. However, it remains unclear whether using websites leads to meaningful and long-lasting behavior change.

**Trial Registration:**

PROSPERO CRD42021283168; https://www.crd.york.ac.uk/prospero/display_record.php?RecordID=283168

## Introduction

### Background

Chronic illness is the leading cause of poor health [1] and disability [2] worldwide. Advances in the medical management of chronic health conditions such as cancer, cardiovascular disease, and stroke have enhanced life expectancy [3,4] but have inadvertently contributed to an increase in the prevalence of chronic health conditions in our community. With people living longer, it is unsurprising that the demand for programs to better manage the effects of chronic illnesses is increasing [5]. One way to reduce the burden of chronic health conditions [6,7] on health care systems is to develop services [5] that assist patients in managing their own modifiable risk factors and improving their quality of life. Common modifiable risk factors [8,9] for chronic health conditions are physical inactivity [10-12] and poor diet [13,14]. Being physically active and eating well when living with a chronic health condition have the potential to improve blood pressure [13], lipid profiles [14], and glucose metabolism [10]. Increasing activity levels may result in lower rates of morbidity and mortality in adults living with chronic health conditions [9]. Given the protective benefits of being physically active and eating well when living with a chronic health condition, it is essential for clinicians and policy makers to consider evidence-based interventions [8] that help prevent and manage chronic illnesses.

Adults living with chronic health conditions experience many challenges in accessing health support services [7,15]. Barriers to accessing in-person chronic health management programs can include long waiting lists, programs not being suitable, difficulty with transportation, and challenges with meeting program costs [15]. Living with a chronic health condition can also result in fatigue [16], impaired function, diminished exercise capacity, and issues with mood and motivation [16]. Furthermore, adults who are disadvantaged or live in a rural setting [16] can be more vulnerable to health-related complications as access to chronic disease management programs is limited. For effective long-term management of chronic health conditions, patients, carers, and health care providers need accessible resources that can support the self-management of modifiable risk factors.

Websites that focus on modifiable risk factor management could potentially address barriers that currently exist in the effective management of chronic health conditions. Data from reviews of individual chronic disease groups such as cancer, cardiovascular disease, and diabetes suggest that self-directed websites could be effective in supporting risk factor management [17-20]. However, a lack of consistency in study design [21], type of interventions, and collected outcomes [21] has made it difficult to pool data into a meta-analysis [18]. No previous reviews have quantified the effect of using websites to help improve physical activity levels or diet quality in adults across a range of chronic health conditions. Given that the challenges in accessing secondary prevention programs [7,22] and the universal goals of treatment across chronic disease groups are similar for adults irrespective of their chronic health condition [22], we chose to undertake this broad review.

### Objectives

The specific research questions for this review were as follows:

1. Is the use of websites containing health information by adults with chronic health conditions effective in improving physical activity or diet quality relative to passive control conditions (no intervention or care as usual) or active control conditions (websites with additional support)?
2. Is the use of websites aimed at improving physical activity or diet quality by adults with chronic health conditions effective in improving self-efficacy or quality of life relative to passive control conditions (no intervention or care as usual) or active control conditions (websites with additional support)?

## Methods

This review is reported according to the PRISMA (Preferred Reporting Items for Systematic Reviews and Meta-Analyses) guidelines (Multimedia Appendix 1 [23]) and was prospectively registered with PROSPERO (CRD42021283168). There were no deviations from the registered protocol for this review.

### Eligibility Criteria

We included randomized controlled trials that tested the effectiveness of using websites designed to provide health information compared with care as usual (eg, waitlist control or no intervention) or with using websites with additional support (eg, supervised by a health professional) on levels of physical activity or diet quality in adults with a chronic health condition [24].

#### Participants

Trials were included if the enrolled participants were adults and had a chronic health condition. We defined *chronic health conditions* as medically diagnosed physical conditions that are long-lasting with persistent effects [8]. Studies with a mix of healthy participants and participants with chronic health conditions were deemed eligible if at least 50% of participants had a chronic health condition as defined by our criteria. We excluded studies testing the effectiveness of interventions on obesity and hypertension as we deemed these to be primary disease markers of a chronic health condition as opposed to a chronic health condition per se. We excluded studies evaluating the effectiveness of interventions for adults with eating disorders, intellectual disabilities, mental health conditions, and complications related to pregnancy, as well as of general lifestyle interventions such as intervention for weight loss.

#### Intervention

For inclusion, trials needed to evaluate the effectiveness of using a website with health information that was not individually tailored to a participant, did not include supervision by or direct contact with a health professional, and could be accessed on any device (eg, desktop computer, laptop, or mobile phone browser). The website needed to promote chronic disease management and had to focus on improving either physical activity levels or diet quality (or both). We included studies in which a generic email or SMS text message was sent to participants as a reminder as long as it was not individualized based on engagement with website content during the intervention period. Contact for technical support from a therapist was acceptable if the therapist did not provide any guidance based on the intervention.

The comparison intervention could be usual care (eg, waitlist control, written pamphlets, or generic website links) or websites with additional behavior change elements (eg, websites with personal tailoring or supervision from a therapist on more than one occasion). Trials were excluded if the intervention was not via a website (eg, SMS text messaging, fitness tracker, console, or phone app), was therapist led (eg, teleconference, face-to-face, or artificial intelligence supported), was not focused on promoting improvements in either diet quality or levels of physical activity (eg, measures of BMI or weight loss), and did not promote chronic disease management (eg, reporting and monitoring symptoms).

#### Outcome Measures

Trials were included if they used standardized self-reported questionnaires for either physical activity or diet quality (eg, International Physical Activity Questionnaire and Diet Quality Index), individual recall methods (eg, exercise tracking sheets or food recall diaries), or device-based data collection (eg, via accelerometers) for physical activity or diet quality. If trials with a primary outcome measure of physical activity or diet quality reported on self-efficacy and quality of life, these data were also extracted.

### Search Strategy

A comprehensive search strategy with the support of an academic librarian was conducted in the following databases—MEDLINE, Embase, CINAHL, and Physiotherapy Evidence Database (PEDro)—from the earliest available record until October 2021. The search was rerun in February 2023 to capture the most recent publications. The search strategy included keywords, Medical Subject Heading terms, and subject headings describing participants (eg, adults with a chronic health condition), the intervention (eg, websites and self-directed), and the outcome of interest (changes to physical activity or diet quality). The search was restricted to studies published in English as restricting it to English-language publications had little impact on the effect estimates and conclusions of this systematic review [25]. The study design was not restricted in the search strategy. The full search strategies are available in Multimedia Appendix 2. To ensure that all possible trials were considered for inclusion, citation tracking was conducted, and the reference lists of all the included trials were also checked. Gray literature was not searched for this review.

### Selection Process

The EndNote X9 (Clarivate Analytics) software was used to manage the titles and abstracts from the individual databases. Once duplicates were removed, all remaining titles and abstracts were exported into Covidence (Veritas Health Innovation) to manage study selection for this review. In total, 2 reviewers (DP and AMD) independently agreed on the selection of eligible studies and achieved consensus on which studies to include by applying the inclusion criteria for the initial search. When the search was rerun in February 2023, a total of 2 reviewers (DP and CE) selected a sample of eligible studies and achieved good agreement with the remainder selected by 1 reviewer (DP). In total, 2 reviewers (DP and AMD) independently reviewed the full texts of all potential titles against the inclusion criteria (Textbox 1). Disagreements or ambiguities were resolved through consensus after discussion with a third reviewer (DBS). Agreement between reviewers was assessed using the κ statistic [26].

**Inclusion criteria.**

**Design**

- Randomized controlled trials published in a peer-reviewed journal in English

**Participants**

- Adults with a chronic health condition

**Intervention**

- Self-directed website
- Aim to promote chronic disease management or survivorship
- Focus on *either* diet and healthy eating habits or physical activity levels

**Outcome measures**

- Physical activity
  Self-efficacy
  Quality of life
- Diet changes
  Self-efficacy
  Quality of life

**Comparisons**

- Usual care
- No intervention
- A more supported web program (eg, supervised)

### Risk-of-Bias Assessment

The methodological quality of the included trials was assessed by 2 reviewers (DP and AMD) independently by applying the PEDro scale [27,28]. This is an 11-item scale comprising 1 item evaluating external validity and 10 items evaluating internal validity. A score of 8 on the PEDro scale is considered the highest possible score as participants and therapists in exercise trials cannot be blinded. The PEDro score demonstrates convergent and construct validity [29] and has moderate levels of interrater reliability (intraclass correlation coefficient=0.68, 95% CI 0.57-0.76) [28]. Disagreements were resolved through consensus. Agreement between reviewers was assessed using the κ statistic [26].

### Data Extraction

At least 2 reviewers achieved a consensus on which data to extract from the included studies. One reviewer (DP) extracted the data, and a second reviewer (DBS) checked them, with final consensus achieved through discussion. Data extracted included participant characteristics (age, gender, and time since diagnosis), type of chronic health condition, type and duration of the intervention, description of comparison group, type of outcomes measured, and results as reported in each individual trial. Where information was not available in the published paper, details were requested from the corresponding authors. In cases in which more than one paper reported data from the same cohort of participants, data from the main trial were included in the analyses.

### Synthesis of Results

Postintervention means and SDs [30] with the corresponding number of participants for outcomes of interest were pooled via meta-analyses using random-effects models. We calculated the weighted mean difference (MD) for meta-analyses that included the same outcome measure and the standardized MD (SMD) where different outcome measures were used to measure the same construct. If data were presented as medians and IQRs, they were converted to means and SDs [31]. If data could not be included in a pooled meta-analysis, the individual trial results were summarized descriptively [32]. Sensitivity and subgroup analyses were performed to assess the robustness of the synthesized results and explore possible causes of heterogeneity [32]. The analyses were performed using Review Manager (version 5.4; The Nordic Cochrane Centre) [33]. For all outcome measures, the critical value for rejecting H0 was set at a level of .05 (2-tailed).

### Publication Bias

**Figure 1:**
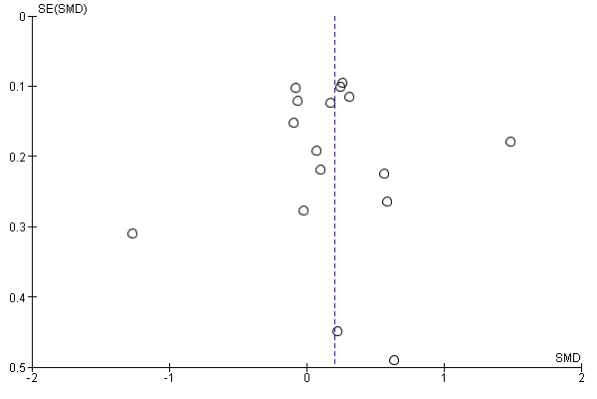
Funnel plot for the physical activity meta-analysis. SMD: standardized mean difference.

Publication bias was assessed using funnel plots for a meta-analysis with >10 included trials. Funnel plots were interpreted through visual inspection of asymmetry. An example of a funnel plot can be found in Figure 1.

### Certainty Assessment

The GRADE (Grading of Recommendations Assessment, Development, and Evaluation) approach was used to summarize the overall certainty of evidence for each outcome [33,34]. The GRADE system specifies 4 levels of certainty for a body of evidence for any given outcome: high, moderate, low, and very low. We downgraded the certainty of evidence by 1 point [35] if one of the following prespecified criteria were present: low methodological quality (defined as >50% of the trials having a PEDro score of <6), inconsistency of estimates among pooled studies (*I*^2^>50%) [34] or effect estimate with wide CIs (ie, CI range of >0.8 SMD), and publication bias suspected when looking at symmetry of funnel plots for a meta-analysis of >10 studies.

## Results

### Study Selection

**Figure 2:**
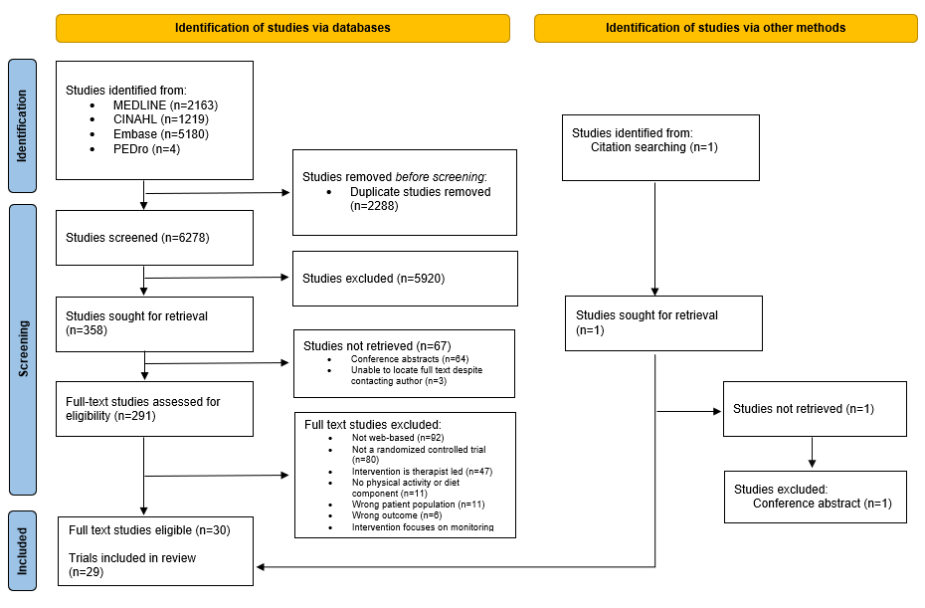
Study selection flowchart. PA: physical activity; PEDro: Physiotherapy Evidence Database.

The electronic database search initially identified 8566 studies, which was reduced to 6278 (73.29%) once duplicate studies were removed. After screening titles, abstracts, and reference lists, 358 full-text studies were retrieved. Substantial agreement (κ=0.73) was achieved between reviewers when screening the titles and abstracts. There were 91.6% (328/358) of studies that failed to meet the inclusion criteria, leaving 30 studies to be included. Moderate agreement (κ=0.51) was achieved between reviewers who screened the full-text papers. The list of excluded studies with reasons for exclusion after full-text screening is available in Multimedia Appendix 3. A total of 7% (2/30) of the studies [36,37] reported data from the same trial; therefore, this systematic review included 30 studies that reported outcomes from 29 trials [36,38-65] (Figure 2).

### Study Characteristics

The 29 trials included 6418 participants across 8 different chronic disease groups (cancer: n=12, 41%; chronic obstructive pulmonary disease: n=1, 3%; cardiovascular disease: n=4, 14%; lower back pain: n=1, 3%; metabolic syndrome: n=1, 3%; type 2 diabetes mellitus: n=7, 24%; stroke: n=2, 7%; and rheumatoid arthritis: n=1, 3%). Most of the included participants were female, with a mean age across all trials of 55 (SD 9) years. A total of 38% (11/29) of the included trials were published in the last 3 years. Additional information was requested from the corresponding authors of 34% (10/29) of the trials and received from those of 7% (2/29) of included trials [38,39]. The study characteristics are described in Table 1.

**Table 1:** Summary of study characteristics and individual results.

Study, year	Country	Condition	Participants	PA^a^ or diet	Program duration	Intervention	Comparison	Outcome measures	Favors the use of websites with health information alone
Chan et al [40], 2020	United States	Cancer	N=202; mean age: 70 (SD 2.9) y; time since diagnosis: mean 5 (SD 2.3) y; female sex: 0%	PA and diet	3 mo	General web-based information (n=49)	Tailored website with feedback (n=51); tailored website+Fitbit+SMS text messaging (n=50); tailored website+Fitbit+SMS text messaging+2 face-to-face sessions (n=52)	MVPA^b^ (min/wk); FFQ^c^; vegetable, processed meat, and fish consumption (serves/wk): self-reported	✗✗✗✗✗^d^
Finlay et al [41], 2020	Australia	Cancer	N=71; mean age: 67 (SD 10) y; time since diagnosis: mean 5.1 (SD 4.4) y; female sex: not reported	PA	4 wk	Nonspecific web links (n=17)	Tunneled modules+ask an expert (n=27); self-paced modules+ask an expert (n=27)	MVPA (min/wk): self-reported GSLTPAQ^e^	✗
Forbes et al [42], 2015	Canada	Cancer	N=95; mean age: 65 (SD 9) y; time since diagnosis: mean 6.6 (SD 2.6) y; female sex: 56%	PA	9 wk	Sequential web modules (n=48)	Usual care (n=47)	MVPA (min/wk): self-reported LSI^f^ from GSLTPAQ; QOL^g^: FACT-G^h^ questionnaire	✗✗
Holtdirk et al [43], 2021	Germany	Cancer	N=363; mean age: 50 (SD 8.2) y; time since diagnosis: not reported; female sex: 100%	PA and diet	3 mo	Website offering cognitive behavioral therapy (n=181)	Waitlist control (n=182)	PA: aerobic METs^i^ (min/wk); diet: food quality questionnaire; QOL: WHOQOL^j^ (brief) total score	✓✓✓
Kanera et al [36,37], 2016 and 2017	The Netherlands	Cancer	N=518; mean age: 56 (SD 11) y; time since diagnosis: mean 24.3 (SD 13.2) wk; female sex: 80%	PA and diet	6-mo and 12-mo follow-up	Self-paced web-based program (n=265)	Waitlist control—with access after 12 mo (n=253)	Moderate PA (min/wk): SQUASH^k^; vegetable consumption (g/d): self-reported	✗✓
Kenfield et al [44], 2019	United States	Cancer	N=76; mean age: 64.8 (SD 1.5) y; time since diagnosis: not reported; female sex: 0%	PA and diet	12 wk	Web program with 4 topics (n=37)	Usual care (n=39)	MVPA (min/wk) and steps (per d) via accelerometer; vegetable, processed meat, and fish consumption (serves/wk): self-reported	✗✓✓✓
Lee et al [45], 2014	South Korea	Cancer	N=59; mean age: 42.4 (SD 5.7) y; time since diagnosis: mean 22.7 (SD 15) wk; female sex: not reported	PA and diet	12 wk	Structured self-paced web program (n=30)	Educational booklet (n=29)	Self-report PA: reported as >150 min/wk (number and percentage); fruit and vegetable 3-d recall: reported as >5 serves/d (number and percentage); DQI^l^: total score; QOL: EORTC QLQ-C30^m^; self-efficacy to increase exercise and fruit and vegetable intake	✓✓✓✗✓
Rabin et al [46], 2011	United States	Cancer	N=18; mean age: 32.2 (SD 5.6) y; time since diagnosis: mean 4.0 (SD 2.5) y; female sex: 56%	PA	12 wk	Website access (n=8)	Web links only (n=10)	MVPA (min/wk): 7-d PAR^n^	✓
Rees-Punia et al [47], 2021	United States	Cancer	N=85; mean age: 60.9 (SD 7.4) y; time since diagnosis: mean 7.0 (SD 1.9) y; female sex: 94%	PA	12 wk	Website access (n=45)	Waitlist control (n=40)	MVPA (min/wk) measured via accelerometer	✗
van Blarigan et al [48], 2020	United States	Cancer	N=50; median age: 55 (IQR 50-62) y; time since diagnosis: median 1.8 (IQR 1-3.3) y; female sex: 66%	Diet	12 wk	Website access (n=25)	Waitlist control (n=25)	Consumption of vegetables, fish, and processed meat (serves/d): 4-d diet records using ASA24^o^	✗✗✗
van de Wiel et al [56], 2021	Switzerland	Cancer	N=137; mean age: 59.8 (SD 11.7) y; time since diagnosis: not reported; female sex: 49%	PA	6 mo	Website access (n=45)	Website+telephone support (n=46); usual care (n=46)	MVPA measured via accelerometer	✗
Yun et al [57], 2020	South Korea	Cancer	N=394; mean age: 53.8 (SD 10.9) y; time since diagnosis: not reported; female sex: 61%	PA	6 mo	Website access (n=125)	Website+health coaching (n=135); usual care (n=134)	MVPA: GSLTPAQ self-reported questionnaire, reported as >150 min/wk (number and percentage)	✗
Wan et al [49], 2017	United States	COPD^p^	N=109; mean age: 53.8 (SD 10.9) y; time since diagnosis: not reported; female sex: 2%	PA	3 mo	Self-paced web program+pedometer (n=57)	Pedometer only (n=52)	PA: step count (pedometer); self-efficacy: Exercise Self-Regulatory Efficacy Scale	✓✗
Antypas and Wangberg [50], 2017	Norway	CVD^q^	N=69; mean age: 59.2 (SD 9.1) y; time since diagnosis: not reported; female sex: 23%	PA	12 wk	Self-directed web-based program (n=28)	Personalized web-based program (n=29)	Moderate PA (min/wk) via IPAQ^r^; self-efficacy: PC-EX^s^ scale	✗✗
Duan et al [38], 2018	China	CVD	N=114; mean age: 48.53 (SD 13.55) y; time since diagnosis: newly diagnosed; female sex: 53%	PA and diet	8 wk (4 wk PA and 4 wk diet)	Weekly website modules (n=60)	Waitlist control (n=54)	Moderate PA (min/wk) via IPAQ; fruit and vegetable consumption, 7-d recall: portions/d (80 g)	✓✓
Engelen et al [58], 2020	The Netherlands	CVD	N=208; mean age: 63.5 (SD 9.9) y; time since diagnosis: mean 4.6 (SD 8.0) y; female sex: 31%	PA and diet	12 mo	Self-paced web program (n=103)	Usual care (n=105)	Moderate PA (min/wk) via IPAQ; Dutch Healthy Diet Index SE Physical Activity and SE Diet; RAND health change questionnaire	✗✗✗✗
Wong et al [59], 2020	China	CVD	N=438; mean age: 52.3 (SD 4.9) y; time since diagnosis: not reported; female sex: 34%	PA	6 mo	Self-paced web-based program (n=219)	Usual care (n=219)	GSLTPAQ; SEE-C^t^ scale	✗✗
Geraghty et al [51], 2018	United Kingdom	LBP^u^	N=87; mean age: 58 (SD 13.5) y; time since diagnosis: not reported; female sex: 61%	PA	6-wk program, follow-up data at 3 mo	Web-based program with 6 modules (n=25)	Web-based program+physiotherapist telephone support (n=22); usual care only (n=26)	Moderate PA (min/wk): self-report IPAQ-SF^v^	✗
Jahangiry et al [60], 2017	Iran	Metabolic syndrome	N=160; mean age: 44.2 (SD 10) y; time since diagnosis: not reported; female sex: 37%	PA and diet	6 mo	Self-paced structured web program (n=80)	General information about risk factors (n=80)	Moderate PA (min/wk): self-reported IPAQ; vegetable intake: self-reported and FFQ; QOL: SF-36^w^ and physical function	✓✓✗
Booth et al [52], 2016	Australia	T2DM^x^	N=84; mean age: 59 (SD 9.9) y; time since diagnosis: not reported; female sex: 42%	PA and diet	12 wk	Self-paced web program (n=41)	List of websites only (n=43)	4-d food diary recording percentage of total energy; moderate PA (min/wk): self-reported IPAQ; QOL: SF-36 and physical function	✗✗✗
Connelly et al [61], 2017	United Kingdom	T2DM	N=31; mean age: 66.7 (SD 8.7) y; time since diagnosis: mean 7.3 (SD 3.7) y; female sex: 42%	PA	6 mo	Information web group (n=10)	Interactive web group (n=11); written T2DM advice (n=10)	MVPA (min/wk) measured via accelerometer; step count (steps/wk)	✗✗✗✗
Glasgow et al [62], 2010	United States	T2DM	N=463; mean age: 58.4 (SD 9.2) y; time since diagnosis: not reported; female sex: 50%	PA and diet	4 mo	Self-paced web-based program (n=169)	Web-based program+social support (n=162); enhanced usual care (n=132)	Fat intake (percentage): Starting The Conversation scale; PA (calories/wk): CHAMPS^y^ questionnaire	✗✗✗
Hansel et al [63], 2017	France	T2DM	N=120; mean age: 56.5 (SD 9.2) y; time since diagnosis: not reported; female sex: 67%	Diet and PA	16 wk	Self-paced web-based program (n=60)	General nutrition advice (n=60)	DQI-I^z^; moderate PA (min/wk): self-report via IPAQ-SF	✓✗
Jennings et al [53], 2014	Australia	T2DM	N=397; mean age: 58.2 (SD 10.3) y; time since diagnosis: mean 8.35 (SD 6.72) y; female sex: 48%	PA	12 wk	Self-paced web-based program with pedometer (n=118)	Pedometer and questionnaire only (n=145)	Moderate PA (min/wk): self-reported IPAQ	✗
Muller et al [54], 2017	United Kingdom, Austria, Germany, Ireland, and Taiwan	T2DM	N=1045; mean age: 62 (SD 9.2) y; time since diagnosis: 9.2 y; female sex: 46%	PA	Immediate pretest-posttest design	Text-only web-based intervention (n=497)	Interactive (tailored) web-based intervention (n=548)	Engagement: self-reported measures of engagement (number of completed sections)	✓
Vluggen et al [64], 2021	The Netherlands	T2DM	N=478; mean age: 60 (SD 6.7) y; time since diagnosis: not reported; female sex: 32%	PA and diet	6 mo	Access to a web-based program (n=234)	Waitlist control (n=244)	Moderate PA (min/wk): SQUASH; self-administered FFQ; snack intake (calories/wk)	✗✓
Guillaumier et al [39], 2022	Australia	Stroke	N=399; mean age: 66 (SD 12) y; time since diagnosis: not reported; female sex: 35%	PA and diet	12 wk	Web-based program (n=199)	Links to generic websites (n=200)	ARFS^aa^; GSLTPAQ	✗✓
Kim et al [55], 2013	South Korea	Stroke	N=36; mean age: 65.6 (SD 7.4) y; time since diagnosis: 3.5 y; female sex: 36%	PA and diet	9 wk	Structured web-based program (n=18)	Standard care (n=18)	Self-reported health behaviors (regular exercise; yes or no); self-efficacy: Mastery Scale; diet: self-reported fruit and vegetable intake (percentage)	✓✓✓
van den Berg et al [65], 2006	The Netherlands	RA^ab^	N=160; median age: 49.6 (IQR 13.4) y; time since diagnosis: 6.6 (10.1) y; female sex: 76%	PA	12 mo	Self-paced web-based program (n=78)	Individually tailored+therapist-guided web-based program (n=82)	PA: moderate or vigorous activity self-reported as number of d/wk physically active; QOL: SF-36 and RAQoL^ac^ score	✗✗

^a^PA: physical activity.

^b^MVPA: moderate to vigorous physical activity.

^c^FFQ: food frequency questionnaire.

^d^A “✓” symbol corresponds to a significant finding as reported by authors of each trial specific to each outcome measure of interest for which data was extracted for this review. An “✗” corresponds to a nonsignificant finding as reported by authors of each trial specific to each outcome measure of interest for which data was extracted for this review and 5 ✗ symbols correspond to 5 such outcomes.

^e^GSLTPAQ: Godin-Shephard Leisure-Time Physical Activity Questionnaire.

^f^LSI: Leisure Score Index.

^g^QOL: quality of life.

^h^FACT-G: Functional Assessment of Cancer Therapy.

^i^MET: metabolic equivalent of task.

^j^WHOQOL: World Health Organization Quality of Life assessment.

^k^SQUASH: Short Questionnaire to Assess Health-Enhancing Physical Activity.

^l^DQI: Diet Quality Index.

^m^EORTC QLQ-C30: European Organization for the Research and Treatment of Cancer Quality of Life Questionnaire.

^n^PAR: Physical Activity Recall.

^o^ASA24: Automatic Self-Administered 24-hour Dietary Assessment Tool.

^p^COPD: chronic obstructive pulmonary disease.

^q^CVD: cardiovascular disease.

^r^IPAQ: International Physical Activity Questionnaire.

^s^PC-EX: Perceived Competence for Regular Physical Exercise.

^t^SEE-C: Chinese version of the Self-Efficacy for Exercise Scale.

^u^LBP: low back pain.

^v^IPAQ-SF: International Physical Activity Questionnaire–Short Form.

^w^SF-36: 36-item Short Form Health Survey.

^x^T2DM: type 2 diabetes mellitus.

^y^CHAMPS: Community Health Activities Model Program for Seniors.

^z^DQI-I: Diet Quality Index-International.

^aa^ARFS: Australian Recommended Food Score.

^ab^RA: rheumatoid arthritis.

^ac^RAQoL: Rheumatoid Arthritis Quality of Life.

The intervention periods ranged from 4 weeks to 12 months. In total, 62% (18/29) of the trials [38-55] included short web interventions (≤3 mo), and 38% (11/29) of the trials [36,56-65] included longer web interventions (>3 mo). A total of 79% (23/29) of the trials [36,38,39,42-49,51-53,55,56,58-64] had a comparator group that was either usual care or a waitlist control (eg, no intervention). In the remaining 21% (6/29) of the trials [40,41,50,54,57,65], the control group was offered use of the same website and additional support (eg, tailored content or supervision from an allied health professional). In total, 14% (4/29) of the trials [51,56,57,61] were multiarm trials with 2 comparator interventions where the use of a website containing health information was compared with usual care as well as with a website with additional support.

### Methodological Quality

The mean PEDro score of the included trials was 5.8 (SD 1.3; Table 2). The agreement of methodological quality ratings between the reviewers was almost perfect (κ=0.901). Most studies reported similar characteristics of the participants across groups at baseline (20/30, 67%) and performed an intention-to-treat analysis (20/30, 67%). Less than half (11/30, 37%) of the included studies had blinded assessors. In 17% (5/30) of the studies, the participants were blinded to the intervention.

**Table 2:** Physiotherapy Evidence Database scores of the included trials (N=29).

Study, year	Random allocation	Concealed allocation	Groups similar at baseline	Participant blinding	Therapist blinding	Assessor blinding	<15% dropout	Intention-to-treat analysis	Between-group difference reported	Point estimate and variability reported	Total (0-10)
Chan et al [40], 2020^a^	1	1	1	1	0	0	0	0	1	1	6
Finlay et al [41], 2020^b^	1	1	0	1	0	0	0	0	1	1	5
Forbes et al [42], 2015^c^	1	0	1	0	0	0	1	1	1	1	6
Holtdirk et al [43], 2021^d^	1	0	1	0	0	0	0	1	1	1	5
Kanera et al [36], 2017^e^	1	1	0	0	0	0	0	1	1	1	5
Kenfield et al [44], 2019^f^	1	1	1	0	0	1	0	0	1	1	6
Lee et al [45], 2014^g^	1	1	1	1	0	0	1	0	1	1	7
Rabin et al [46], 2011^h^	1	0	0	0	0	0	1	1	0	1	4
Rees-Punia et al [47], 2021^i^	1	0	1	0	0	1	1	0	0	1	5
van Blarigan et al [48], 2020^j^	1	1	0	0	0	0	1	0	1	1	5
van de Wiel et al [56], 2021^k^	1	1	1	0	0	0	0	1	1	1	6
Yun et al [57], 2020^l^	1	0	1	0	0	1	0	1	1	1	6
Wan et al [49], 2017^m^	1	1	1	0	0	1	1	1	1	1	8
Antypas and Wangberg [50], 2017^n^	1	0	0	1	0	1	0	0	1	1	5
Duan et al [38], 2018^o^	1	0	1	0	0	0	0	0	1	1	4
Engelen et al [58], 2020^p^	1	0	1	0	0	0	1	1	1	1	6
Wong et al [59], 2020^q^	1	1	1	0	0	1	1	1	1	1	8
Geraghty et al [51], 2018^r^	1	1	1	0	0	1	0	1	1	1	7
Jahangiry et al [60], 2017^s^	1	0	1	0	0	0	1	1	1	1	6
Booth et al [52], 2016^t^	1	0	1	0	0	0	0	0	1	1	4
Connelly et al [61], 2017^u^	1	1	0	0	0	0	1	0	0	1	4
Glasgow et al [62], 2010^v^	1	0	1	0	0	0	0	1	1	1	5
Hansel et al [63], 2017^w^	1	0	1	0	0	1	1	1	1	1	7
Jennings et al [53], 2014^x^	1	1	0	0	0	0	0	1	1	1	5
Muller et al [54], 2017^y^	1	1	0	1	0	1	0	1	1	1	7
Vluggen et al [64], 2021^z^	1	0	0	0	0	0	0	1	1	1	4
Guillaumier et al [39], 2022^aa^	1	1	1	0	0	1	1	1	1	1	8
Kim et al [55], 2013^ab^	1	0	1	0	0	0	1	1	1	1	6
van den Berg et al [65], 2006^ac^	1	1	1	0	0	1	1	1	1	1	8

^a^Project supported by the National Center for Advancing Translational Sciences, National Institutes of Health (NIH), grants UL1 TR001872 and UL1TR002369, and other NIH grants (P30DK098722, P30 CA069533-21, and National Cancer Institute K07CA197077).

^b^Amy Finlay was supported by a PhD scholarship provided by the University of Adelaide as well as a scholarship top-up by the Freemasons Centre for Male Health. Project costs were covered by a start-up package provided to Camile E Short when commencing at the Freemasons Centre for Male Health. Camile E Short was also supported by a National Health and Medical Research Council Early Career Research fellowship.

^c^This work was supported by the Canada Research Chairs Program held by Kerry Courneya.

^d^This study was funded by Gaia, Germany, a research-focused small to medium enterprise that specializes in eHealth interventions and regularly participates in publicly funded research. There was no external funding.

^e^Research project funded by the Dutch Cancer Society (grant NOU2011-5151).

^f^This work was supported by grants from the Prostate Cancer Foundation; the American Cancer Society; and the Department of Urology, University of California, San Francisco.

^g^Authors declared no funding source.

^h^This research was funded by an award from the National Cancer Institute (R03 CA134197).

^i^The American Cancer Society funds the creation, maintenance, and updating of the Cancer Prevention Study–3.

^j^The research reported in this publication was supported by the National Cancer Institute of the NIH under award K07CA197077 to EVB.

^k^This project (NKI 2015-7904) is funded by the Dutch Cancer Society (*KWF Kankerbestrijding*). The funding body had no role in the design of the study; collection, analysis, and interpretation of the data; or writing of the manuscript.

^l^This study was supported by grants from the National R&D Program for Cancer Control, Ministry of Health and Welfare, Republic of Korea (1320330), and partially by a grant from the Korea Health Technology R&D Project through the Korea Health Industry Development Institute, funded by the Ministry of Health and Welfare, Republic of Korea (grant HC15C1391).

^m^Department of Veterans Affairs Office of Rehabilitation Research and Development Service (Career Development Award 2, F6847W [MM]; Career Development Award 2, IK2RX002165 [EW]; Merit O1150-R [MM]).

^n^Fully funded by a PhD grant from the Northern Norway Regional Health Authority (ID 3342/HST986-10).

^o^Research supported by a junior research group grant from the *Wilhelm-Stiftung für Rehabilitationsforschung* in Germany and a faculty research grant from Hong Kong Baptist University in Hong Kong (FRG1/12-13/064).

^p^This study was funded by ZonMw, the Netherlands Organization for Health Research and Development (520001001).

^q^This research was supported by the Health and Medical Research Fund (10110301), the Food and Health Bureau, and the Hong Kong Special Administrative Regions.

^r^This paper presents independent research funded by the National Institute for Health and Care Research (NIHR) for Patient Benefit program (PB-PG-1111-26080). Nadine E Foster and Jonathan C Hill are funded by an NIHR Research Professorship awarded to Nadine E Foster (NIHR-RP-011-015), and Lisa C Roberts is funded by a Higher Education Funding Council for England and National Institute of Health and Care Research Senior Clinical Lectureship (round 3). Nadine E Foster, Elaine M Hay, and Paul Little are National Institute of Health and care Research senior investigators.

^s^This manuscript originated from a PhD thesis (240/2425) by LJ.

^t^Source of funding not reported by the study authors.

^u^Source of funding not reported by the study authors.

^v^Supported by grant DK35524 from the National Institute of Diabetes and Digestive and Kidney Diseases.

^w^The Medical Expert Systems-MXS company kindly provided the MXS-CARE e-coaching program for use in this study. The company did not take part in the data analyses.

^x^Corneel Vandelanotte was supported by the National Health and Medical Research Council of Australia (519778) and a National Heart Foundation of Australia (PH 07B 3303) postdoctoral research fellowship.

^y^This research was part of the Diabetes Literacy project supported by grant FP7-Health-2012-Innovation-1/306186 of the European Commission.

^z^This study was supported by the Maastricht University Medical Center+ Strategy Horizon 2020. The funding source had no involvement in conducting the research or preparing the research paper.

^aa^This study was funded by a grant from the National Health and Medical Research Council (NHMRC; APP1125429) awarded to Billie Bonevski, Neil J Spratt, Michael Pollack, Amanda Baker, Parker Magin, Alyna Turner, Christopher Oldmeadow, Clare Collins, and Robin Callister. Ashleigh Guillaumier was supported by a postdoctoral fellowship from the National Heart Foundation (award 101303). Neil J Spratt was supported by a cofunded Australian NHMRC and National Heart Foundation Career Development/Future Leader Fellowship (GNT1110629/100827). Amanda Baker was supported by an NHMRC Research Fellowship (G1200044). The funders had no role in the study design, data collection and analysis, decision to publish, or preparation of the manuscript.

^ab^This work was supported by the Basic Science Research Program through the National Research Foundation of Korea funded by the Ministry of Education, Science, and Technology (20110003345).

^ac^Supported by ZonMw (Netherlands Organization for Health Research and Development; grant 4010.0004) and the *Reumafonds* (Dutch Arthritis Foundation).

### Effect of Using Websites Containing Health Information Compared With Usual Care on Physical Activity Levels

#### Overview

**Figure 3:**
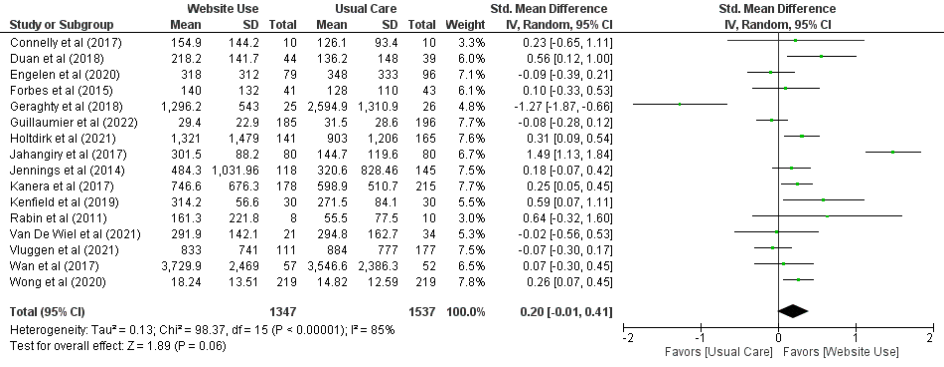
Standardized mean difference (95% CI) of the effect of using websites containing health information compared with usual care on physical activity levels by pooling data from 16 trials.

A total of 79% (23/29) of the trials compared the effect of the use of websites containing health information with that of usual care on physical activity levels. A meta-analysis of 55% (16/29) of the trials (Figure 3 [37-39,42-44,46,49,51,53,56,58-61,64]) including 2884 participants found no between-group differences in physical activity levels when comparing the use of websites that contain health information with usual care (SMD=0.20, 95% CI −0.01 to 0.41). A sensitivity analysis was performed to remove 6% (1/16) of these trials [51] with implausibly high baseline levels of physical activity. A meta-analysis of the remaining 94% (15/16) of the trials including 2833 participants found moderate-certainty evidence with a small effect size that the use of websites containing health information increased physical activity levels in adults living with chronic health conditions (SMD=0.27, 95% CI 0.08-0.46) when compared with usual care (Table 3).

**Figure 4:**
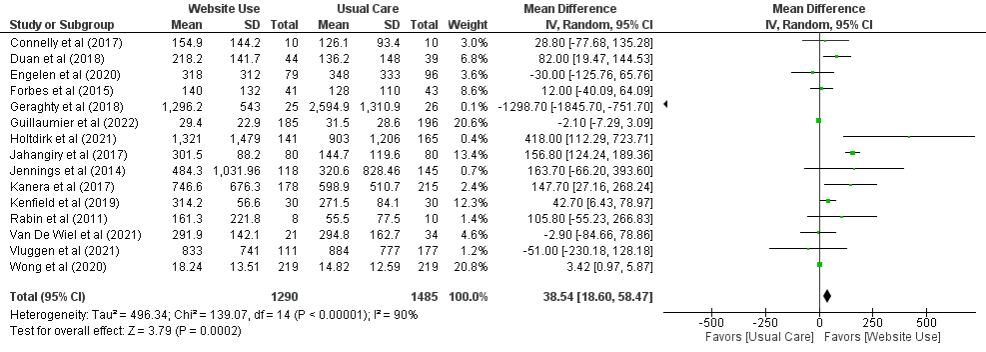
Mean difference (95% CI) of the effect of using websites containing health information compared with usual care on time spent in moderate to vigorous physical activity by pooling data from 15 trials.

When a meta-analysis of 52% (15/29) of the trials (Figure 4 [37-39,42-44,46,49,51,53,56,58-61,64]) including 2775 participants was performed on reported physical activity levels as minutes of moderate to vigorous physical activity (self-reported and device based) per week, there was moderate-certainty evidence that using websites containing health information increased time spent in moderate to vigorous physical activity when compared with usual care (MD=39 min/wk, 95% CI 18.60-58.47; Table 3).

Data from 7% (2/29) of the trials [45,55] on the proportion of participants who met the recommended 150 minutes per week of moderate physical activity could not be pooled for meta-analysis. In one trial [45] (N=59), the authors found that 66% of participants in the intervention group compared with 36% in the usual care group (*P*<.001) achieved the recommended physical activity levels. In the other trial [55] (N=394), there were no between-group differences for participants who met physical activity recommendations (*P*=.08).

**Table 3:** Overall meta-analyses of the effects of using websites containing health information on physical activity levels, quality of life, self-efficacy, and diet changes.

Outcome and comparison group		Trials (n=29), n (%)	Participants	MD^a^ (95% CI)	SMD^b^ (95% CI)	Quality of evidence (GRADE^c^)
**Physical activity^d^**						
	Usual care	16 (55)	2884	N/A^e^	0.20 (−0.01 to 0.41)	Moderate^f^
	Usual care^g^	15 (52)	2833	N/A	0.27 (0.08 to 0.46)	Moderate^f^
	Website+additional support	7 (24)	572	N/A	0.14 (−0.26 to 0.54)	Low^h^
**MVPA^i^ (min/wk)**						
	Website+additional support^j^	2 (7)	67	46.97 (−21.16 to 115.10)	N/A	Moderate^k^
**Processed meat consumption (serves/wk)**						
	Usual care	2 (7)	109	1.14 (0.70 to 1.58)	N/A	High
**Healthy diet^l^**						
	Usual care	4 (14)	958	N/A	0.13 (−0.11 to 0.37)	Moderate^k^
**Vegetable intake (serves/wk)**						
	Usual care	5 (17)	755	N/A	0.47 (−0.01 to 0.94)	Very low^m^
**Fish consumption (serves/wk)**						
	Usual care	2 (7)	109	0.86 (−0.86 to 2.57)	N/A	Low^n^
**Quality of life^o^**						
	Usual care	6 (21)	877	N/A	0.36 (0.12 to 0.59)	Moderate^f^
**Self-efficacy^p^**						
	Usual care	4 (14)	1693	N/A	0.26 (0.05 to 0.48)	Moderate^f^

^a^MD: mean difference.

^b^SMD: standardized mean difference.

^c^GRADE: Grading of Recommendations Assessment, Development, and Evaluation. *Working group grades of evidence*: high quality (further research is very unlikely to change our confidence in the estimate of the effect), moderate quality (further research is likely to have an important impact on our confidence in the estimate of the effect and may change the estimate), low quality (further research is very likely to have an important impact on our confidence in the estimate of the effect and is likely to change the estimate), and very low quality (we are very uncertain about the estimate).

^d^*Physical activity (PA)*: questionnaires reporting on moderate to vigorous PA levels in minutes per week (eg, International Physical Activity Questionnaire, Community Health Activities Model Program for Seniors, Godin-Shephard Leisure-Time Physical Activity Questionnaire, and Short Questionnaire to Assess Health-Enhancing Physical Activity), self-reported active minutes per week, and device-measured PA levels (eg, accelerometer), as well as steps per day.

^e^N/A: not applicable.

^f^Reason for downgrade: *I*^2^ score of >50%.

^g^Sensitivity analysis to remove trials with high median metabolic equivalent of task minutes of PA at baseline, and the authors declared that trial participants likely substantially overestimated their activity using the International Physical Activity Questionnaire–Short Form tool.

^h^Reason for downgrade: *I*^2^ score of >50% *and* >50% of trials with a Physiotherapy Evidence Database (PEDro) score of <6.

^i^MVPA: moderate to vigorous physical activity.

^j^Objectively measured moderate to vigorous physical activity.

^k^Reason for downgrade: 95% CI range of >0.8 standardized mean difference or >2 times the mean difference.

^l^*Diet quality:* questionnaires reporting on diet quality (eg, Dutch Healthy Diet Index, Diet Quality Index, food frequency questionnaire, or self-reported consumption of fruit or vegetables or processed meat or fish or legumes in either serves per d or per wk or either yes or no response to meeting guidelines).

^m^Reason for downgrade: *I*^2^ score of >50% *and* >50% of trials with a PEDro score of <6 *and* 95% CI range of >0.8 standardized mean difference or >2 times the mean difference.

^n^Reason for downgrade: *I*^2^ score of >50% *and* 95% CI range of >0.8 standardized mean difference or >2 times the mean difference.

^o^*Quality of life*: a variety of questionnaires were used (eg, health change questionnaire, quality of life Functional Assessment of Cancer Therapy, World Health Organization Quality of Life–Brief, and *RAND*-SF36).

^p^*Self-efficacy:* a variety of methods were used to report on self-efficacy (eg, Self-Management Ability–Patient Activation Measure, Chinese version of the Self-Efficacy for Exercise Scale, self-reported satisfaction, and the Mastery Scale).

#### Subgroup Analysis: Effect of Using Websites Containing Health Information by Disease Type

Subgroup analyses were performed for specific disease groups. For adults living with cancer, a meta-analysis of 21% (6/29) of the trials [36,42-44,46,56] including 916 participants found high-certainty evidence in favor of using a website containing health information for improving physical activity levels (MD=55 min/wk, 95% CI 1.60-107.40) compared with usual care. For adults with type 2 diabetes [53,61,64] or cardiovascular disease [38,58,59], no between-group differences were found for increasing physical activity levels when comparing the use of websites containing health information with usual care (Table 4).

**Table 4:** Subgroup meta-analyses of the effect of using websites containing health information on physical activity levels by outcome measurement, program duration, and chronic health condition.

Outcome	Comparison group	Trials (n=29), n (%)	Participants	MD^a^ (95% CI)	SMD^b^ (95% CI)	Quality of evidence (GRADE^c^)
Physical activity: MVPA^d^ only^e^	Usual care	15 (52)	2775	38.54 (18.60 to 58.47)	N/A^f^	Moderate^g^
Physical activity: device based only^e^	Usual care	3 (10)	135	34.63 (2.98 to 66.29)	N/A	High
Physical activity: program duration (<3 mo)	Usual care	9 (31)	1355	N/A	0.12 (−0.13 to 0.37)	Moderate^g^
Physical activity: program duration (>3 mo)^e^	Usual care	7 (24)	1529	41.38 (−33.80 to 116.56)	N/A	Low^h^
Physical activity: cancer^e^	Usual care	6 (21)	916	54.50 (1.60 to 107.40)	N/A	High
Physical activity: T2DM^e,i^	Usual care	3 (10)	571	29.57 (−58.6 to 117.8)	N/A	Low^j^
Physical activity: CVD^e,k^	Usual care	3 (10)	696	20.64 (−34.88 to 76.16)	N/A	Low^l^

^a^MD: mean difference.

^b^SMD: standardized mean difference.

^c^GRADE: Grading of Recommendations Assessment, Development, and Evaluation.

^d^MVPA: moderate to vigorous physical activity.

^e^All mean differences are measured in minutes per week.

^f^N/A: not applicable.

^g^Reason for downgrade: *I*^2^ score of >50%.

^h^Reason for downgrade: *I*^2^ score of >50% and 95% CI range of >0.8 standardized mean difference or >2 times the mean difference.

^i^T2DM: type 2 diabetes mellitus.

^j^Reason for downgrade: 95% CI range of >0.8 standardized mean difference or >2 times the mean difference and >50% of trials with a PEDro score of <6.

^k^CVD: cardiovascular disease.

^l^Reason for downgrade: *I*^2^ score of >50% and >50% of trials with a PEDro score of <6 and 95% CI range of >0.8 standardized mean difference or >2 times the mean difference.

#### Subgroup Analysis: Effect of Using Websites Containing Health Information by Intervention Duration

When the trials were grouped into short (<3 mo) and long (≥3 mo) interventions and compared with usual care, no between-group differences were found (Table 4).

#### Subgroup Analysis: Effect of Using Websites Containing Health Information Compared With Usual Care on Processed Meat Consumption

**Figure 5:**
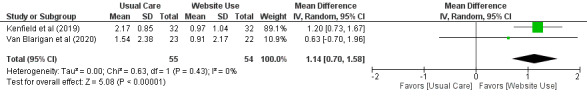
Mean difference (95% CI) of the effect of using websites containing health information compared with usual care on reducing processed meat consumption by pooling data from 2 trials.

A meta-analysis of 7% (2/29) of the trials including 109 participants found high-certainty evidence that using websites containing health information reduced processed meat consumption for adults living with chronic health conditions (MD=1.14 serves/wk, 95% CI 0.70-1.58; Figure 5 [44,48]) when compared with usual care.

#### Subgroup Analysis: Effect of Using Websites Containing Health Information Compared With Usual Care on Other Diet Measures

Data from 7% (2/29) of the included trials [52,64] could not be combined for meta-analysis because of heterogeneity in outcomes. One trial [64] found reduced snack intake in calories per week when accessing self-directed websites compared with usual care (*P*=.002). The second trial [52] did not find any between-group differences in consumption of protein (*P*=.54), carbohydrates (*P*=.06), sugars (*P*=.71), sodium (*P*=.38), or fiber (*P*=.29) when comparing the use of websites containing health information with usual care.

There were no between-group differences in healthy diet questionnaire scores or vegetable and fish consumption when comparing groups that used websites containing health information with usual care (Table 3).

#### Subgroup Analysis: Effect of Using Websites Containing Health Information Compared With Usual Care on Quality of Life

**Figure 6:**
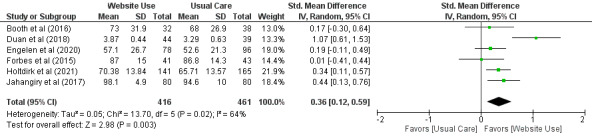
Standardized mean difference (95% CI) of the effect of using websites containing health information compared with usual care on quality of life by pooling data from 6 trials. Questionnaires used: health change questionnaire, quality of life Functional Assessment of Cancer Therapy, World Health Organization Quality of Life–Brief, and RAND-36.

A meta-analysis of 21% (6/29) of the trials including 877 participants found moderate-certainty evidence with a small to moderate effect size that using websites containing health information improved quality of life in adults with chronic health conditions (SMD=0.36, 95% CI 0.12-0.59; Figure 6 [38,42,43,52,58,60]) when compared with usual care.

#### Subgroup Analysis: Effect of Using Websites Containing Health Information Compared With Usual Care on Self-Efficacy

**Figure 7:**
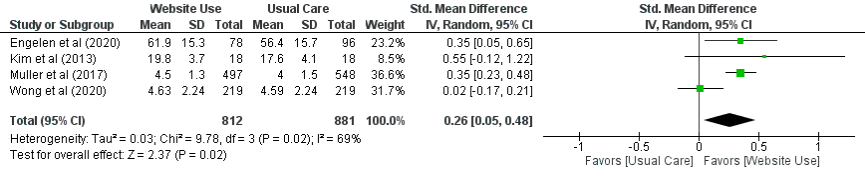
Standardized mean difference (95% CI) of the effect of using websites containing health information compared with usual care on self-efficacy by pooling data from 4 trials. Questionnaires used: Self-Management Ability–Patient Activation Measure, Chinese version of the Self-Efficacy for Exercise Scale, self-reported satisfaction, and the Mastery Scale.

A meta-analysis of 14% (4/29) of the trials including 1693 participants found moderate-certainty evidence with a small effect size that using websites containing health information improved self-efficacy in adults living with chronic health conditions (SMD=0.26, 95% CI 0.05-0.48; Figure 7 [54,55,58,59]) when compared with usual care.

#### Subgroup Analysis: Effect of Using Websites Containing Health Information Compared With Using the Same Websites With Additional Support on Physical Activity Levels

A total of 34% (10/29) of the trials compared the effect of using websites containing health information with that of using the same websites offering additional support on physical activity levels. A meta-analysis of 70% (7/10) of these trials including 572 participants found no between-group differences in physical activity minutes (SMD=0.14, 95% CI –0.26 to 0.54) when comparing the effect of the use of websites containing health information with that of the use of the same websites offering additional support on improving physical activity minutes.

#### Subgroup Analysis: Effect of Using Websites Containing Health Information Compared With Using the Same Websites With Additional Support on Other Outcomes of Interest

No between-group differences were found for outcomes related to diet quality, self-efficacy, and quality of life when comparing the use of websites containing health information with the use of websites with additional support.

## Discussion

### Principal Findings

In this systematic review and meta-analysis, we found moderate- to high-certainty evidence with small to moderate effect sizes that using websites that contain health information improved physical activity levels, quality of life, and self-efficacy in adults living with chronic health conditions. There was also high-certainty evidence that using websites containing health information may lead to reduced processed meat consumption. No substantial effects were found for other dietary outcomes in favor of using websites over usual care. Furthermore, our results demonstrate no added benefit of personalized support when using websites that contain health information and promote self-management in adults living with chronic health conditions. This review adds to the literature as the first to evaluate the effect of using websites that contain health information on behavior change in terms of physical activity levels and diet quality for risk factor management in a broad range of adults living with a variety of chronic health conditions.

Using websites that contain health information may result in clinically meaningful improvements in physical activity levels for adults living with chronic health conditions. Our findings are consistent with those of previous reviews suggesting that websites that contain health information have the potential to effectively support adults with cancer, diabetes, and cardiovascular disease [17-20,66] in improving their levels of activity. The estimated mean increase of 39 active minutes per week observed after using websites aimed at increasing physical activity equates to >25% of the recommended weekly physical activity as per current guidelines [67]. An estimated mean increase of 35 active minutes per week was observed when physical activity was measured using a device, further strengthening our main findings by removing the potential for self-reporting bias. The findings of our review confirm, with moderate to high certainty, that the use of websites that contain health information can lead to significant and clinically meaningful improvements in levels of physical activity for adults living with chronic health conditions.

Using websites that contain health information may lead to a reduction in processed meat consumption but not to increases in fruit, vegetable, or fish intake. Diet patterns that are high in processed meat [68,69] are associated with an increased risk of developing type 2 diabetes, cancer, and cardiovascular disease [70], so a demonstrated reduction in intake with the use of websites that contain health information is an important finding. Diet patterns that are high in vegetables, fruits, fish, and legumes have a protective effect against chronic disease [69,70], yet we were unable to demonstrate an increase in the consumption of these with the use of websites that contain health information. It is possible that the use of these types of websites is better able to induce a reduction in the consumption of *risk type* foods (eg, processed meats) as opposed to an increase in the consumption of *protective* foods (eg, vegetables). It is also possible that any beneficial effects are diluted by trying to induce simultaneous changes in multiple dietary factors (eg, increases in the consumption of fruits, vegetables, fish, and legumes simultaneously) [71]. However, we acknowledge that our inability to demonstrate the effectiveness of using websites that contain health information in increasing “protective” food consumption was likely affected by the heterogeneity associated with how the consumption of these foods was measured. Trials variously measured foods in grams, serves (defined in multiple ways), and frequencies, and this made it difficult to draw any strong conclusions from pooled data on the effectiveness of websites with health information to improve these aspects of diet [72]. Nevertheless, using such websites appears likely to have a positive influence on decreasing processed meat intake, and it may be that, if websites targeted increased intakes of only 1 or 2 “positive” foods, significant changes could be achieved. Consistency in how the consumption of these foods is measured, defined, and reported in the literature will help researchers understand the true impacts of websites with health information on behavior change that results in diet changes for adults living with chronic health conditions.

Web-based interventions may be effective in enhancing quality of life and improving self-efficacy in adults living with chronic health conditions [73,74]. Building self-efficacy early in the chronic disease process empowers adults living with chronic illnesses to take greater control of their health [74]. Key elements to successful risk factor management programs include having access to disease-specific knowledge and suitable tools for self-management [75]. Therefore, changes in physical activity and dietary behavior through the use of websites with health information may be mediated by improvements in self-efficacy through information provision. Our findings suggest that carefully designed websites that contain health information are well placed to provide users with disease-specific information and motivation to build their capacity for behavior change in relation to physical activity and diet quality [76].

Challenges accessing suitable in-person risk factor management programs continue to exist for adults living with chronic health conditions, especially in rural and regional settings [77]. The COVID-19 pandemic has led to the rapid and widespread uptake of digital technology with improvements in infrastructure and greater user confidence worldwide [78]. Therefore, the provision of health information via websites as described in this study may help overcome issues related to lack of in-person care to support positive changes in the lifestyle behaviors of adults living with chronic health conditions. It appears that web-based interventions could be a solution to the access gap that currently exists between adults with chronic health conditions living in rural and metropolitan areas [79], but it should be acknowledged that differences in health literacy [80] as well as differences in access to appropriate broadband and technology may affect the ability of adults living rurally to benefit from this type of intervention [81]. Despite the potential for some issues that could arise from upscaling the use of websites with health information, it seems appropriate to consider whether such an intervention could be a low-resource way to reach and support more adults in managing their chronic health conditions irrespective of where they live and what support they have around them [81].

### Strengths and Limitations

This review has several strengths. Our review of 29 trials included participants living with a variety of chronic health conditions across many countries worldwide, increasing the generalizability of our results. Our review was prospectively registered and synthesized very recent evidence (11/29, 38% of the included trials were published in the last 3 y), making the findings relevant to current issues faced in health care settings. The methodology used in this review was robust and with careful appraisal of quality of evidence via the GRADE approach, with most meta-analysis (7/10, 70%) rated as “moderate” or “high” in certainty of evidence, increasing the confidence in the estimate of the effect as seen in our results.

It is important to note that this review has some limitations. One limitation was heterogeneity and inconsistency in the reporting of diet data, which made it difficult to draw meaningful conclusions on the effectiveness of using websites with health information on behavior change in relation to diet quality. However, in the absence of more objective data, an evaluation of the best available evidence can still inform practice. For measures we could pool, we used SMD to account for the variability in how diet quality was measured or reported. Although there did not appear to be any added benefit of providing additional support when using websites that contain health information, these findings should be interpreted with caution because of the small number of included trials with these comparisons in this review. The same can be said for findings such as a reduction in processed meat consumption, which were obtained from a small sample. Similarly, our findings in relation to quality of life and self-efficacy are specific to trials that also looked at physical activity or diet quality and, therefore, may have missed other trials specifically looking at these outcomes. It should also be acknowledged that we could not isolate which specific website components contributed to effectiveness (eg, content and interface design), and therefore, we are unable to make any recommendations on what individual elements in websites that contain health information have the greatest effect. We are also unable to draw any conclusions about whether using websites that contain health information results in sustained behavior change as this was not the purpose of this review. Finally, participants who agreed to take part in the research were likely to be motivated and ready for change compared with those who chose not to take part in the research. This may affect the generalizability of our findings to patient cohorts who experience challenges with engagement and may not yet be ready for change [82].

### Conclusions

The use of websites that contain health information can lead to some healthy lifestyle changes in adults living with chronic health conditions, improve levels of physical activity, reduce processed meat consumption, and improve self-efficacy and quality of life. Providing access to disease-specific websites for chronic disease management may offer a realistic alternative to no intervention for patients who have challenges adopting positive lifestyle behaviors, particularly if they are unable to access health care providers for more intensive therapies. Further research is warranted to explore which specific features of websites have the greatest effect and whether using websites with health information can lead to long-lasting behavior change. To further our understanding of the type of person who stands to benefit most from using websites to promote behavior change in relation to physical activity or diet quality, we recommend that future trials focus on accounting for variables such as age, gender, severity of chronic health condition, and digital competence.

## Appendix

Multimedia Appendix 1 PRISMA (Preferred Reporting Items for Systematic Reviews and Meta-Analyses) 2020 checklist.

Multimedia Appendix 2 Full search strategies.

Multimedia Appendix 3 Table of excluded papers.

## Abbreviations

GRADE: Grading of Recommendations Assessment, Development, and Evaluation
MD: mean difference
PEDro: Physiotherapy Evidence Database
PRISMA: Preferred Reporting Items for Systematic Reviews and Meta-Analyses
SMD: standardized mean difference

## Data Availability

The data sets analyzed during this study are available from the corresponding author upon reasonable request.
