# Supplementary material for: Effects of Using Websites on Physical Activity and Diet Quality for Adults Living With Chronic Health Conditions: Systematic Review and Meta-Analysis
Source: J Med Internet Res. 2023 Oct 19;25:e49357. doi: 10.2196/49357 (PMC10623240; doi:10.2196/49357)
Supplement: Multimedia Appendix 2 [file jmir_v25i1e49357_app2.docx]

Appendix 2. Search strategies

Medline.

| **#** | **Terms** | **Results** |
| --- | --- | --- |
| 1 | Telemedicine/ | 36,822 |
| 2 | Information Technology/ | 785 |
| 3 | Online Systems/ | 8,545 |
| 4 | Mobile Applications/ | 11,254 |
| 5 | Computers, Handheld/ | 4,055 |
| 6 | Fitness Trackers/ | 1,112 |
| 7 | (technolog* or computer* or telephone* or telemed* or tele med* or telehealth or tele health or telerehab* or ict or electronic health or ehealth or e health or e-health or online or on-line or internet or website or web-site or web platform or web-platform or web based or web-based or mobile health or mhealth or m-health or digi* or short message service or sms or text messag* or multimedia messaging service or mms or app or apps or mobile application or smartphone or smart phone or mobile phone).ti. | 371,942 |
| 8 | 1 or 2 or 3 or 4 or 5 or 6 or 7 | 396,138 |
| 9 | exp Stroke/ | 170,455 |
| 10 | exp Chronic Disease/ | 618,433 |
| 11 | NEOPLASMS/ | 496,145 |
| 12 | cardiovascular diseases/ | 175,199 |
| 13 | DIABETES MELLITUS/ | 137,410 |
| 14 | ARTHRITIS/ | 37,081 |
| 15 | ASTHMA/ | 138,662 |
| 16 | Obesity/ | 213,935 |
| 17 | Weight Gain/ | 35,344 |
| 18 | exp Weight Loss/ | 48,802 |
| 19 | (weight gain or weight loss or weight change*).ti. | 29,785 |
| 20 | ((bmi or body mass index) adj2 (gain or loss or change)).ti. | 470 |
| 21 | Back Pain/ | 18,905 |
| 22 | exp Pulmonary Disease, Chronic Obstructive/ | 66,289 |
| 23 | (cvd or cardiovascular disease* or coronary artery disease or cancer* or neoplasm* or diabet* or asthma* or back pain or obes* or arthrit* or copd or chronic obstructive or stroke or heart dis*).ti. | 2,368,999 |
| 24 | ((chronic* or persistent or long* term or ongoing or degenerative) adj3 (disease* or disab* or ill* or condition*)).ti. | 113,984 |
| 25 | (obes* adj2 (prevent* or treat*)).ti. | 7,230 |
| 26 | 9 or 10 or 11 or 12 or 13 or 14 or 15 or 16 or 17 or 18 or 19 or 20 or 21 or 22 or 23 or 24 or 25 | 3,630,118 |
| 27 | exp Exercise/ or exp Exercise Therapy/ | 280,403 |
| 28 | Rehabilitation/ | 18,679 |
| 29 | (physical education or physical training or physical exercis* or physical therap* or physiotherap* or physical inactiv* or physical activ*).ti. | 79,232 |
| 30 | Physical Fitness/ | 29,680 |
| 31 | Physical Therapy Modalities/ | 40,607 |
| 32 | exp Life Style/ | 109,973 |
| 33 | (((lifestyle* or life style*) adj5 (program* or intervent* or activ* or change*)) or secondary prevent*).ti. | 11,118 |
| 34 | 27 or 28 or 29 or 30 or 31 or 32 or 33 | 468,736 |
| 35 | exp Diet/ | 326,459 |
| 36 | (healthy eat* or eat well or eat for heath or nutrition).ti. | 72,049 |
| 37 | Feeding Behavior/ or ((feeding or eating) adj behavio?r*).ti. | 93,879 |
| 38 | (dietary intake or diet* intervent* or diet* change* or diet modif*).ti. | 7,950 |
| 39 | Food Habits/ | 92,332 |
| 40 | Dietary Fats, Unsaturated/ or Dietary Fats/ | 54,096 |
| 41 | ((feeding or food or nutrition*) adj program*).ti. | 1,436 |
| 42 | 35 or 36 or 37 or 38 or 39 or 40 or 41 | 485,201 |
| 43 | 34 or 42 | 910,549 |
| 44 | (intervention* or therap* or program* or treatment*).tw. | 8,644,540 |
| 45 | 8 and 26 and 43 and 44 | 2,207 |
| **46** | **limit 45 to English language** | **2,163** |

Embase.

| **#** | **Terms** | **Results** |
| --- | --- | --- |
| 1 | Telemedicine/ | 43,755 |
| 2 | Information Technology/ | 13,495 |
| 3 | Online Systems/ | 29,726 |
| 4 | Mobile Applications/ | 19,937 |
| 5 | Computers, Handheld/ | 1,784 |
| 6 | Fitness Trackers/ | 2,064 |
| 7 | (technolog* or computer* or telephone* or telemed* or tele med* or telehealth or tele health or telerehab* or ict or electronic health or ehealth or e health or e-health or online or on-line or internet or website or web-site or web platform or web-platform or web based or web-based or mobile health or mhealth or m-health or digi* or short message service or sms or text messag* or multimedia messaging service or mms or app or apps or mobile application or smartphone or smart phone or mobile phone).ti. | 464,014 |
| 8 | 1 or 2 or 3 or 4 or 5 or 6 or 7 | 522,943 |
| 9 | exp Stroke/ | 310,218 |
| 10 | exp Chronic Disease/ | 232,144 |
| 11 | NEOPLASMS/ | 92,248 |
| 12 | cardiovascular diseases/ | 113,419 |
| 13 | DIABETES MELLITUS/ | 731,031 |
| 14 | ARTHRITIS/ | 89,283 |
| 15 | ASTHMA/ | 287,457 |
| 16 | Obesity/ | 545,391 |
| 17 | Weight Gain/ | 115,385 |
| 18 | exp Weight Loss/ | 83,747 |
| 19 | (weight gain or weight loss).ti. | 38,999 |
| 20 | weight change*.ti. | 3,444 |
| 21 | ((bmi or body mass index) adj2 (gain or loss or change)).ti. | 697 |
| 22 | Back Pain/ | 24,414 |
| 23 | exp Pulmonary Disease, Chronic Obstructive/ | 173,735 |
| 24 | (cvd or cardiovascular disease* or coronary artery disease or cancer* or neoplasm* or diabet* or asthma* or back pain or obes* or arthrit* or copd or chronic obstructive or stroke or heart dis*).ti. | 3,426,365 |
| 25 | ((chronic* or persistent or long* term or ongoing or degenerative) adj3 (disease* or disab* or ill* or condition*)).ti. | 157,209 |
| 26 | (obes* adj2 (prevent* or treat*)).ti. | 9,736 |
| 27 | 9 or 10 or 11 or 12 or 13 or 14 or 15 or 16 or 17 or 18 or 19 or 20 or 21 or 22 or 23 or 24 or 25 or 26 | 5,011,997 |
| 28 | exp Exercise/ or exp Exercise Therapy/ | 507,708 |
| 29 | Rehabilitation/ | 113,739 |
| 30 | physical inactiv*.ti. | 1,438 |
| 31 | physical activ*.ti. | 64,071 |
| 32 | (physical education or physical training or physical exercis* or physical therap* or physiotherap*).ti. | 39,293 |
| 33 | Physical Fitness/ | 40,611 |
| 34 | Physical Therapy Modalities/ | 107,632 |
| 35 | exp Life Style/ | 164,033 |
| 36 | (((lifestyle* or life style*) adj5 (program* or intervent* or activ* or change*)) or secondary prevent*).ti. | 16,238 |
| 37 | 28 or 29 or 30 or 31 or 32 or 33 or 34 or 35 or 36 | 894,217 |
| 38 | exp Diet/ | 449,648 |
| 39 | nutrition*.ti. | 153,837 |
| 40 | (healthy eat* or eat well or eat for heath).ti. | 2,461 |
| 41 | Feeding Behavior/ or ((feeding or eating) adj behavio?r*).ti. | 101,096 |
| 42 | (dietary intake or diet* intervent* or diet* change* or diet modif*).ti. | 10,248 |
| 43 | Food Habits/ | 83,405 |
| 44 | Dietary Fats, Unsaturated/ or Dietary Fats/ | 55,939 |
| 45 | ((feeding or food or nutrition*) adj program*).ti. | 1,782 |
| 46 | 38 or 39 or 40 or 41 or 42 or 43 or 44 or 45 | 698,702 |
| 47 | 37 or 46 | 1,525,506 |
| 48 | (intervention* or therap* or program* or treatment*).tw. | 12,430,021 |
| 49 | 8 and 27 and 47 and 48 | 5,250 |
| **50** | **limit 49 to english language** | **5,180** |

CINAHL.

|  | Terms | Result |
| --- | --- | --- |
| **S42** | **S40 AND S41** | **1,219** |
| S41 | (randomized controlled trials OR MH double-blind studies OR MH single-blind studies OR MH random assignment OR MH pretest-posttest design OR MH cluster sample OR TI (randomised OR randomized) OR AB (random*) OR TI (trial) OR (MH (sample size) AND AB (assigned OR allocated OR control)) OR MH (placebos) OR PT (randomized controlled trial) OR AB (control W5 group) OR MH (crossover design) OR MH (comparative studies) OR AB (cluster W3 RCT)) NOT ((MH animals+ OR MH animal studies OR TI animal model*) NOT MH human) | 943,536 |
| S40 | S8 AND S24 AND S38 AND S39 | 2,135 |
| S39 | TI ( intervention* or therap* or program* or treatment* ) OR AB ( intervention* or therap* or program* or treatment* ) | 2,108,860 |
| S38 | S25 OR S26 OR S27 OR S28 OR S29 OR S30 OR S31 OR S32 OR S33 OR S34 OR S35 OR S36 OR S37 | 648,288 |
| S37 | (MH "Fats, Unsaturated") OR (MH "Dietary Fats") | 14,989 |
| S36 | TI dietary intake or diet* intervent* or diet* change* or diet modif* | 9,727 |
| S35 | (MH "Food Habits") | 16,325 |
| S34 | (MH "Eating Behavior") | 20,556 |
| S33 | TI healthy eat* or eat* well or eat* for heath or nutrition | 43,757 |
| S32 | (MH "Diet+") | 137,812 |
| S31 | TI secondary prevention | 2,504 |
| S30 | (MH "Life Style+") | 268,672 |
| S29 | (MH "Physical Therapy") | 37,900 |
| S28 | (MH "Physical Fitness") | 19,223 |
| S27 | TI physical education or physical training or physical exercis* or physical therap* or physiotherap* or physical inactiv* or physical activ* | 60,434 |
| S26 | (MH "Rehabilitation") | 19,420 |
| S25 | (MH "Exercise+") OR (MH "Therapeutic Exercise") | 147,178 |
| S24 | S9 OR S10 OR S11 OR S12 OR S13 OR S14 OR S15 OR S16 OR S17 OR S18 OR S19 OR S20 OR S21 OR S22 OR S23 | 1,044,947 |
| S23 | TI cvd or cardiovascular disease* or coronary artery disease or cancer* or neoplasm* or diabet* or asthma* or back pain or obes* or arthrit* or copd or chronic obstructive or stroke or heart dis*) | 760,868 |
| S22 | (MH "Pulmonary Disease, Chronic Obstructive+") | 22,065 |
| S21 | (MH "Back Pain") | 11,910 |
| S20 | TI (bmi or body mass index) adj2 (gain or loss or change) | 0 |
| S19 | TI weight gain or weight loss or weight change | 16,595 |
| S18 | (MH "Weight Loss+") | 26,227 |
| S17 | (MH "Weight Gain") | 13,550 |
| S16 | (MH "Obesity") | 92,964 |
| S15 | (MH "Asthma") | 37,033 |
| S14 | (MH "Arthritis") | 11,403 |
| S13 | (MH "Diabetes Mellitus") | 72,236 |
| S12 | (MH "Cardiovascular Diseases") | 59,018 |
| S11 | (MH "Neoplasms") | 93,544 |
| S10 | (MH "Chronic Disease+") | 72,875 |
| S9 | (MH "Stroke+") | 78,830 |
| S8 | S1 OR S2 OR S3 OR S4 OR S5 OR S6 OR S7 | 229,512 |
| S7 | TI technolog* or computer* or telephone* or telemed* or tele med* or telehealth or tele health or telerehab* or ict or electronic health or ehealth or e health or e-health or online or on-line or internet or website or web-site or web platform or web-platform or web based or web-based or mobile health or mhealth or m-health or digi* or short message service or sms or text messag* or multimedia messaging service or mms or app or apps or mobile application or smartphone or smart phone or mobile phone | 203,562 |
| S6 | (MH "Fitness Trackers") | 358 |
| S5 | (MH "Computers, Hand-Held") OR (MH "Computers, Portable") | 5,908 |
| S4 | (MH "Mobile Applications") | 11,966 |
| S3 | (MH "Online Systems") | 1,892 |
| S2 | (MH "Information Technology") | 14,579 |
| S1 | (MH "Telerehabilitation") OR (MH "Telemedicine") | 16,463 |
