# Supplementary material for: Effects of Using Websites on Physical Activity and Diet Quality for Adults Living With Chronic Health Conditions: Systematic Review and Meta-Analysis
Source: J Med Internet Res. 2023 Oct 19;25:e49357. doi: 10.2196/49357 (PMC10623240; doi:10.2196/49357)
Supplement: Multimedia Appendix 3 [file jmir_v25i1e49357_app3.docx]

**Appendix 3**: Excluded papers (n=328)

| Papers |  | | |  | | Reasons for exclusion | | | | | |  |  |
| --- | --- | --- | --- | --- | --- | --- | --- | --- | --- | --- | --- | --- | --- |
|  | **1** | **2** | **3** | | **4** | | **5** | **6** | **7** | **8** | **9** | **10** | **11** |
| Adu 2020 | ✓ |  |  | |  | |  |  |  |  |  |  |  |
| Adu 2018 | ✓ |  |  | |  | |  |  |  |  |  |  |  |
| Akinci (2017 |  |  | ✓ | |  | |  |  |  |  |  |  |  |
| Algeo 2015 |  |  | ✓ | |  | |  |  |  |  |  |  |  |
| Alonso-Domínguez 2019 | ✓ |  |  | |  | |  |  |  |  |  |  |  |
| Ammendola 2017 |  |  | ✓ | |  | |  |  |  |  |  |  |  |
| An 2017 |  |  | ✓ | |  | |  |  |  |  |  |  |  |
| Aneni 2018 |  | ✓ |  | |  | |  |  |  |  |  |  |  |
| Antypas 2012 |  | ✓ |  | |  | |  |  |  |  |  |  |  |
| Ariza-Garcia 2019 |  |  |  | | ✓ | |  |  |  |  |  |  |  |
| Ayre 2019 | ✓ |  |  | |  | |  |  |  |  |  |  |  |
| Baer 2020 |  |  | ✓ | |  | |  |  |  |  |  |  |  |
| Bannell 2022 |  |  | ✓ | |  | |  |  |  |  |  |  |  |
| Bantum 2014 |  |  |  | | ✓ | |  |  |  |  |  |  |  |
| Barberan-Garcia 2014 |  |  |  | | ✓ | |  |  |  |  |  |  |  |
| Barnes 2016 |  | ✓ |  | |  | |  |  |  |  |  |  |  |
| Barradell 2018 |  | ✓ |  | |  | |  |  |  |  |  |  |  |
| Bender 2018 |  | ✓ |  | |  | |  |  |  |  |  |  |  |
| Bennett 2010 |  |  |  | | ✓ | |  |  |  |  |  |  |  |
| Blanton 2018 |  |  |  | |  | |  |  | ✓ |  |  |  |  |
| Boekhout 2018 |  | ✓ |  | |  | |  |  |  |  |  |  |  |
| Boels 2019 | ✓ |  |  | |  | |  |  |  |  |  |  |  |
| Bonn 2018 | ✓ |  |  | |  | |  |  |  |  |  |  |  |
| Bosak 2007 |  | ✓ |  | |  | |  |  |  |  |  |  |  |
| Bosworth 2018 |  |  | ✓ | |  | |  |  |  |  |  |  |  |
| Boudreau 2011 | ✓ |  |  | |  | |  |  |  |  |  |  |  |
| Bradway 2019 |  |  | ✓ | |  | |  |  |  |  |  |  |  |
| Broers 2020 | ✓ |  |  | |  | |  |  |  |  |  |  |  |
| Burkow 2018 | ✓ |  |  | |  | |  |  |  |  |  |  |  |
| Burkow 2013 |  |  |  | | ✓ | |  |  |  |  |  |  |  |
| Burnett 1987 |  |  | ✓ | |  | |  |  |  |  |  |  |  |
| Buscemi 2020 | ✓ |  |  | |  | |  |  |  |  |  |  |  |
| Cardol 2022 |  |  |  | | ✓ | |  |  |  |  |  |  |  |
| Carruthers 2013 |  |  | ✓ | |  | |  |  |  |  |  |  |  |
| Cerdan 2017 |  | ✓ |  | |  | |  |  |  |  |  |  |  |
| Chaplin 2017 |  |  |  | | ✓ | |  |  |  |  |  |  |  |
| Chen 2016 |  | ✓ |  | |  | |  |  |  |  |  |  |  |
| Cheong 2018 | ✓ |  |  | |  | |  |  |  |  |  |  |  |
| Choi 2018 |  |  | ✓ | |  | |  |  |  |  |  |  |  |
| Cipolla 2020 |  | ✓ |  | |  | |  |  |  |  |  |  |  |
| Claes 2019 |  |  | ✓ | |  | |  |  |  |  |  |  |  |
| Compare 2012 | ✓ |  |  | |  | |  |  |  |  |  |  |  |
| Connelly 2015 |  | ✓ |  | |  | |  |  |  |  |  |  |  |
| Conroy 2012 |  | ✓ |  | |  | |  |  |  |  |  |  |  |
| Coulter 2015 |  |  | ✓ | |  | |  |  |  |  |  |  |  |
| Coulter 2015 |  | ✓ |  | |  | |  |  |  |  |  |  |  |
| Dack 2019 |  | ✓ |  | |  | |  |  |  |  |  |  |  |
| Dale 2014 | ✓ |  |  | |  | |  |  |  |  |  |  |  |
| Dallabrida 2017 |  | ✓ |  | |  | |  |  |  |  |  |  |  |
| Daud 2020 |  | ✓ |  | |  | |  |  |  |  |  |  |  |
| Davies 2012 |  | ✓ |  | |  | |  |  |  |  |  |  |  |
| Dekker-vanWeering 2016 |  |  |  | | ✓ | |  |  |  |  |  |  |  |
| delPozo-Cruz 2013 |  |  |  | |  | | ✓ |  |  |  |  |  |  |
| Denham 2018 |  |  | ✓ | |  | |  |  |  |  |  |  |  |
| Denham 2019 |  | ✓ |  | |  | |  |  |  |  |  |  |  |
| Denham 2018 |  | ✓ |  | |  | |  |  |  |  |  |  |  |
| Denham 2018 |  |  |  | |  | |  |  |  |  |  |  | ✓ |
| Dennis 2016 |  |  | ✓ | |  | |  |  |  |  |  |  |  |
| Devine 2020 | ✓ |  |  | |  | |  |  |  |  |  |  |  |
| Diamond 2021 | ✓ |  |  | |  | |  |  |  |  |  |  |  |
| Doddaiah 2020 | ✓ |  |  | |  | |  |  |  |  |  |  |  |
| Evans 2020 |  | ✓ |  | |  | |  |  |  |  |  |  |  |
| Evans 2021 |  |  |  | | ✓ | |  |  |  |  |  |  |  |
| Evans 2018 |  |  | ✓ | |  | |  |  |  |  |  |  |  |
| Felker 2022 | ✓ |  |  | |  | |  |  |  |  |  |  |  |
| Ferrante 2017 |  |  | ✓ | |  | |  |  |  |  |  |  |  |
| Fico 2020 |  |  |  | |  | |  |  |  |  | ✓ |  |  |
| Forbes 2017 |  |  |  | |  | | ✓ |  |  |  |  |  |  |
| Frensham 2014 |  | ✓ |  | |  | |  |  |  |  |  |  |  |
| Frensham 2020 |  |  |  | | ✓ | |  |  |  |  |  |  |  |
| Frensham 2018 |  |  |  | | ✓ | |  |  |  |  |  |  |  |
| Fuemmeler 2020 |  |  |  | |  | |  |  |  | ✓ |  |  |  |
| Galdiz 2021 |  |  |  | | ✓ | |  |  |  |  |  |  |  |
| Galiano-Castillo 2013 |  | ✓ |  | |  | |  |  |  |  |  |  |  |
| Galiano-Castillo 2016 |  |  |  | | ✓ | |  |  |  |  |  |  |  |
| Gao 2022 | ✓ |  |  | |  | |  |  |  |  |  |  |  |
| GarthMcKay 2002 |  |  |  | | ✓ | |  |  |  |  |  |  |  |
| Geraghty 2020 |  |  |  | |  | |  |  | ✓ |  |  |  |  |
| Geraghty 2015 |  | ✓ |  | |  | |  |  |  |  |  |  |  |
| Girouard 2017 |  |  | ✓ | |  | |  |  |  |  |  |  |  |
| Glasgow 2006 | ✓ |  |  | |  | |  |  |  |  |  |  |  |
| Glasgow 2011 |  | ✓ |  | |  | |  |  |  |  |  |  |  |
| Glasgow 2012 |  |  |  | |  | |  | ✓ |  |  |  |  |  |
| Glasgow 2006 | ✓ |  |  | |  | |  |  |  |  |  |  |  |
| Golsteijn 2013 |  |  | ✓ | |  | |  |  |  |  |  |  |  |
| Golsteijn 2017 |  | ✓ |  | |  | |  |  |  |  |  |  |  |
| Golsteijn 2014 |  |  | ✓ | |  | |  |  |  |  |  |  |  |
| Golsteijn 2017 |  | ✓ |  | |  | |  |  |  |  |  |  |  |
| Golsteijn 2018 |  |  |  | | ✓ | |  |  |  |  |  |  |  |
| Goyal 2016 | ✓ |  |  | |  | |  |  |  |  |  |  |  |
| Grau-Pellicer 2020 | ✓ |  |  | |  | |  |  |  |  |  |  |  |
| Grau-Pellicer 2020 | ✓ |  |  | |  | |  |  |  |  |  |  |  |
| Groarke 2019 |  | ✓ |  | |  | |  |  |  |  |  |  |  |
| Gupta 2020 | ✓ |  |  | |  | |  |  |  |  |  |  |  |
| Haberlin 2018 |  |  | ✓ | |  | |  |  |  |  |  |  |  |
| Haberlin 2020 | ✓ |  |  | |  | |  |  |  |  |  |  |  |
| Hakala 2021 |  |  |  | | ✓ | |  |  |  |  |  |  |  |
| Hallward 2020 |  | ✓ |  | |  | |  |  |  |  |  |  |  |
| Hauser 1992 |  | ✓ |  | |  | |  |  |  |  |  |  |  |
| Henshall 2020 | ✓ |  |  | |  | |  |  |  |  |  |  |  |
| Hewitt 2015 |  |  | ✓ | |  | |  |  |  |  |  |  |  |
| Hidrus 2022 | ✓ |  |  | |  | |  |  |  |  |  |  |  |
| Hirschey 2018 | ✓ |  |  | |  | |  |  |  |  |  |  |  |
| Hochsmann 2017 | ✓ |  |  | |  | |  |  |  |  |  |  |  |
| Hofmann 2016 |  |  |  | | ✓ | |  |  |  |  |  |  |  |
| Holtdirk 2020 |  | ✓ |  | |  | |  |  |  |  |  |  |  |
| Hong 2021 | ✓ |  |  | |  | |  |  |  |  |  |  |  |
| Hong 2015 | ✓ |  |  | |  | |  |  |  |  |  |  |  |
| Hoogstad 2019 |  |  | ✓ | |  | |  |  |  |  |  |  |  |
| Hou 2020 | ✓ |  |  | |  | |  |  |  |  |  |  |  |
| Houchen-Wolloff 2018 |  |  | ✓ | |  | |  |  |  |  |  |  |  |
| Huang 2021 |  |  |  | |  | |  |  | ✓ |  |  |  |  |
| Hurkmans 2010 |  |  |  | |  | |  |  |  |  |  |  | ✓ |
| Hwang 2017 |  |  | ✓ | |  | |  |  |  |  |  |  |  |
| Idris 2020 |  |  |  | | ✓ | |  |  |  |  |  |  |  |
| Isaacson 2012 |  |  |  | |  | |  |  |  |  |  | ✓ |  |
| Jahangiry 2015 |  |  |  | |  | |  |  |  |  |  |  | ✓ |
| Jenny 2001 |  |  |  | |  | | ✓ |  |  |  |  |  |  |
| Jensen 2014 |  | ✓ |  | |  | |  |  |  |  |  |  |  |
| Ji 2019 | ✓ |  |  | |  | |  |  |  |  |  |  |  |
| Jia 2020 |  | ✓ |  | |  | |  |  |  |  |  |  |  |
| Jiang 2020 | ✓ |  |  | |  | |  |  |  |  |  |  |  |
| Jiang 2013 |  | ✓ |  | |  | |  |  |  |  |  |  |  |
| Johansson 2022 |  |  |  | | ✓ | |  |  |  |  |  |  |  |
| Johnston 2009 |  |  |  | |  | |  |  |  |  | ✓ |  |  |
| Jolly 2017 |  |  | ✓ | |  | |  |  |  |  |  |  |  |
| Jolly 2016 |  |  | ✓ | |  | |  |  |  |  |  |  |  |
| Jones 2014 |  |  | ✓ | |  | |  |  |  |  |  |  |  |
| Kar 2020 | ✓ |  |  | |  | |  |  |  |  |  |  |  |
| Karimi 2020 |  | ✓ |  | |  | |  |  |  |  |  |  |  |
| Kelley 2009 |  |  | ✓ | |  | |  |  |  |  |  |  |  |
| Kenfield 2018 |  |  | ✓ | |  | |  |  |  |  |  |  |  |
| Kerr 2010 |  | ✓ |  | |  | |  |  |  |  |  |  |  |
| Khurana 2015 |  |  |  | |  | |  |  |  |  |  | ✓ |  |
| Kim 2013 |  | ✓ |  | |  | |  |  |  |  |  |  |  |
| Kim 2015 | ✓ |  |  | |  | |  |  |  |  |  |  |  |
| Kim 2017 | ✓ |  |  | |  | |  |  |  |  |  |  |  |
| Kim 2020 |  | ✓ |  | |  | |  |  |  |  |  |  |  |
| Kim 2013 | ✓ |  |  | |  | |  |  |  |  |  |  |  |
| Kobe 2020 |  |  |  | | ✓ | |  |  |  |  |  |  |  |
| Kooiman 2018 |  |  |  | | ✓ | |  |  |  |  |  |  |  |
| Koot 2019 | ✓ |  |  | |  | |  |  |  |  |  |  |  |
| Krebs 2017 |  |  | ✓ | |  | |  |  |  |  |  |  |  |
| Krusche 2019 |  | ✓ |  | |  | |  |  |  |  |  |  |  |
| Krylov 2017 |  | ✓ |  | |  | |  |  |  |  |  |  |  |
| Laakso 2011 |  |  | ✓ | |  | |  |  |  |  |  |  |  |
| Lacaille 2015 |  |  | ✓ | |  | |  |  |  |  |  |  |  |
| Lacaille 2016 |  |  | ✓ | |  | |  |  |  |  |  |  |  |
| Lambert 2022 |  | ✓ |  | |  | |  |  |  |  |  |  |  |
| Leach 2019 |  |  |  | |  | |  |  | ✓ |  |  |  |  |
| Lee 2020 |  |  | ✓ | |  | |  |  |  |  |  |  |  |
| Lee 2013 |  | ✓ |  | |  | |  |  |  |  |  |  |  |
| Lelieveld 2011 |  |  |  | |  | |  |  |  | ✓ |  |  |  |
| Lelieveld 2010 |  |  |  | |  | |  |  |  | ✓ |  |  |  |
| Li 2021 | ✓ |  |  | |  | |  |  |  |  |  |  |  |
| Liacos 2018 |  |  |  | | ✓ | |  |  |  |  |  |  |  |
| Liebreich 2009 |  |  |  | | ✓ | |  |  |  |  |  |  |  |
| Lim 2018 | ✓ |  |  | |  | |  |  |  |  |  |  |  |
| Lim 2020 |  |  | ✓ | |  | |  |  |  |  |  |  |  |
| Lindsay 2009 | ✓ |  |  | |  | |  |  |  |  |  |  |  |
| Liu 2019 | ✓ |  |  | |  | |  |  |  |  |  |  |  |
| Lobelo 2016 | ✓ |  |  | |  | |  |  |  |  |  |  |  |
| Look 2016 |  | ✓ |  | |  | |  |  |  |  |  |  |  |
| Lorig 2008 |  |  |  | | ✓ | |  |  |  |  |  |  |  |
| Lorig 2010 |  |  |  | | ✓ | |  |  |  |  |  |  |  |
| Lozano-Lozano 2022 | ✓ |  |  | |  | |  |  |  |  |  |  |  |
| Lygidakis 2019 | ✓ |  |  | |  | |  |  |  |  |  |  |  |
| Lynch 2017 |  |  |  | | ✓ | |  |  |  |  |  |  |  |
| Lystrup 2020 | ✓ |  |  | |  | |  |  |  |  |  |  |  |
| Lyu 2021 |  |  |  | | ✓ | |  |  |  |  |  |  |  |
| Macdonald 2020 |  |  |  | | ✓ | |  |  |  |  |  |  |  |
| Macdonald 2020 |  |  |  | | ✓ | |  |  |  |  |  |  |  |
| Maddison 2015 | ✓ |  |  | |  | |  |  |  |  |  |  |  |
| Mahmood 2013 |  | ✓ |  | |  | |  |  |  |  |  |  |  |
| Majithia 2020 |  | ✓ |  | |  | |  |  |  |  |  |  |  |
| Mazzotti 2014 |  |  | ✓ | |  | |  |  |  |  |  |  |  |
| McCarroll 2015 | ✓ |  |  | |  | |  |  |  |  |  |  |  |
| McKay 2001 |  |  |  | | ✓ | |  |  |  |  |  |  |  |
| Mensorio 2019 |  |  |  | |  | |  |  |  | ✓ |  |  |  |
| Monteiro-Guerra 2020 | ✓ |  |  | |  | |  |  |  |  |  |  |  |
| Moreau 2015 |  | ✓ |  | |  | |  |  |  |  |  |  |  |
| Morrison 2016 |  |  |  | |  | |  |  | ✓ |  |  |  |  |
| Moy 2012 |  |  |  | |  | |  |  |  |  | ✓ |  |  |
| Mumcu 2022 |  |  |  | |  | |  |  | ✓ |  |  |  |  |
| Munteanu 2020 | ✓ |  |  | |  | |  |  |  |  |  |  |  |
| Murray 2017 |  |  |  | |  | | ✓ |  |  |  |  |  |  |
| Muscat 2020 | ✓ |  |  | |  | |  |  |  |  |  |  |  |
| Nero 2018 |  |  | ✓ | |  | |  |  |  |  |  |  |  |
| Nguyen 2013 |  |  |  | | ✓ | |  |  |  |  |  |  |  |
| Nichols 2019 | ✓ |  |  | |  | |  |  |  |  |  |  |  |
| Nielsen 2020 | ✓ |  |  | |  | |  |  |  |  |  |  |  |
| Noormohammadpour 2021 |  |  |  | |  | |  |  |  | ✓ |  |  |  |
| Nurwanti 2019 |  |  |  | |  | |  |  |  |  |  | ✓ |  |
| Nyberg 2017 |  | ✓ |  | |  | |  |  |  |  |  |  |  |
| O'Shea 2020 | ✓ |  |  | |  | |  |  |  |  |  |  |  |
| Ormel 2018 | ✓ |  |  | |  | |  |  |  |  |  |  |  |
| Pan 2011 |  |  | ✓ | |  | |  |  |  |  |  |  |  |
| Park 2019 | ✓ |  |  | |  | |  |  |  |  |  |  |  |
| Park 2016 | ✓ |  |  | |  | |  |  |  |  |  |  |  |
| Patnaik 2022 | ✓ |  |  | |  | |  |  |  |  |  |  |  |
| Paxton 2014 | ✓ |  |  | |  | |  |  |  |  |  |  |  |
| Peel 2020 |  | ✓ |  | |  | |  |  |  |  |  |  |  |
| Pekmezi 2021 |  | ✓ |  | |  | |  |  |  |  |  |  |  |
| Pemu 2011 |  |  |  | |  | | ✓ |  |  |  |  |  |  |
| Pierce 2012 |  | ✓ |  | |  | |  |  |  |  |  |  |  |
| Pihlajamaki 2019 |  | ✓ |  | |  | |  |  |  |  |  |  |  |
| Plow 2017 | ✓ |  |  | |  | |  |  |  |  |  |  |  |
| Poppe 2018 |  | ✓ |  | |  | |  |  |  |  |  |  |  |
| Poppe 2019 |  | ✓ |  | |  | |  |  |  |  |  |  |  |
| Poppe 2019 |  | ✓ |  | |  | |  |  |  |  |  |  |  |
| Post 2016 |  | ✓ |  | |  | |  |  |  |  |  |  |  |
| Poulsen 2016 |  |  | ✓ | |  | |  |  |  |  |  |  |  |
| Puszkiewicz 2016 | ✓ |  |  | |  | |  |  |  |  |  |  |  |
| Quinn 2012 | ✓ |  |  | |  | |  |  |  |  |  |  |  |
| Rafiq 2021 |  |  |  | | ✓ | |  |  |  |  |  |  |  |
| Ramadas 2018 |  |  |  | |  | | ✓ |  |  |  |  |  |  |
| Ramadas 2015 |  | ✓ |  | |  | |  |  |  |  |  |  |  |
| Rassouli 2018 | ✓ |  |  | |  | |  |  |  |  |  |  |  |
| Reed 2005 |  | ✓ |  | |  | |  |  |  |  |  |  |  |
| Reid 2012 |  |  |  | | ✓ | |  |  |  |  |  |  |  |
| Revenas 2014 |  |  | ✓ | |  | |  |  |  |  |  |  |  |
| Reynolds 2018 |  | ✓ |  | |  | |  |  |  |  |  |  |  |
| Ribeiro 2017 | ✓ |  |  | |  | |  |  |  |  |  |  |  |
| Ridad 2020 | ✓ |  |  | |  | |  |  |  |  |  |  |  |
| Rimmer 2013 | ✓ |  |  | |  | |  |  |  |  |  |  |  |
| Ritvo 2017 |  | ✓ |  | |  | |  |  |  |  |  |  |  |
| Robertson 2007 |  |  |  | |  | |  |  |  |  | ✓ |  |  |
| Robertson 2017 | ✓ |  |  | |  | |  |  |  |  |  |  |  |
| Robinson 2021 |  |  |  | | ✓ | |  |  |  |  |  |  |  |
| Robinson 2020 |  |  | ✓ | |  | |  |  |  |  |  |  |  |
| Robinson 2019 |  |  |  | | ✓ | |  |  |  |  |  |  |  |
| Robinson 2020 |  |  |  | |  | |  |  |  |  | ✓ |  |  |
| Rochette 2008 |  |  |  | |  | |  |  | ✓ |  |  |  |  |
| Rossen 2020 |  |  |  | |  | |  |  | ✓ |  |  |  |  |
| Sadler 2017 |  | ✓ |  | |  | |  |  |  |  |  |  |  |
| Sakakibara 2022 | ✓ |  |  | |  | |  |  |  |  |  |  |  |
| Sakakibara 2018 |  | ✓ |  | |  | |  |  |  |  |  |  |  |
| Sakane 2016 | ✓ |  |  | |  | |  |  |  |  |  |  |  |
| Salsman 2020 |  |  |  | |  | |  |  |  | ✓ |  |  |  |
| Sankaran 2019 |  |  |  | | ✓ | |  |  |  |  |  |  |  |
| Sano 2016 |  |  | ✓ | |  | |  |  |  |  |  |  |  |
| Sarfati 2018 |  | ✓ |  | |  | |  |  |  |  |  |  |  |
| Saslow 2017 | ✓ |  |  | |  | |  |  |  |  |  |  |  |
| Schweier 2014 |  |  | ✓ | |  | |  |  |  |  |  |  |  |
| Schweier 2018 |  | ✓ |  | |  | |  |  |  |  |  |  |  |
| Schweier 2014 |  | ✓ |  | |  | |  |  |  |  |  |  |  |
| Seven 2022 | ✓ |  |  | |  | |  |  |  |  |  |  |  |
| Sevick 2018 | ✓ |  |  | |  | |  |  |  |  |  |  |  |
| Shah 2011 |  | ✓ |  | |  | |  |  |  |  |  |  |  |
| Sherifali 2012 |  | ✓ |  | |  | |  |  |  |  |  |  |  |
| Short 2014 |  |  |  | |  | |  |  |  |  |  |  | ✓ |
| Short 2017 |  | ✓ |  | |  | |  |  |  |  |  |  |  |
| Short 2017 |  |  |  | |  | |  | ✓ |  |  |  |  |  |
| Silarova 2019 |  |  |  | |  | |  |  |  | ✓ |  |  |  |
| Singh 2017 |  |  | ✓ | |  | |  |  |  |  |  |  |  |
| Smith 2018 |  |  | ✓ | |  | |  |  |  |  |  |  |  |
| Spielmanns 2020 | ✓ |  |  | |  | |  |  |  |  |  |  |  |
| St-Jules 2022 |  |  |  | | ✓ | |  |  |  |  |  |  |  |
| Steinert 2020 | ✓ |  |  | |  | |  |  |  |  |  |  |  |
| Stenlund 2019 |  | ✓ |  | |  | |  |  |  |  |  |  |  |
| Stine 2022 | ✓ |  |  | |  | |  |  |  |  |  |  |  |
| Stubbins 2017 |  |  | ✓ | |  | |  |  |  |  |  |  |  |
| SuprajaSankaran 2018 |  | ✓ |  | |  | |  |  |  |  |  |  |  |
| Sureshkumar 2015 |  | ✓ |  | |  | |  |  |  |  |  |  |  |
| Sylvia 2023 |  |  |  | |  | |  |  |  | ✓ |  |  |  |
| Sztym 2018 |  |  | ✓ | |  | |  |  |  |  |  |  |  |
| Tabak 2013 |  | ✓ |  | |  | |  |  |  |  |  |  |  |
| Tabak 2014 |  |  |  | | ✓ | |  |  |  |  |  |  |  |
| Tandon 2022 |  | ✓ |  | |  | |  |  |  |  |  |  |  |
| Taylor 2020 |  |  |  | |  | |  |  |  | ✓ |  |  |  |
| Taylor 2021 |  |  |  | |  | |  |  |  | ✓ |  |  |  |
| Teriö 2019 | ✓ |  |  | |  | |  |  |  |  |  |  |  |
| Tian 2021 |  |  | ✓ | |  | |  |  |  |  |  |  |  |
| Timmerman 2015 |  | ✓ |  | |  | |  |  |  |  |  |  |  |
| Timmerman 2017 |  |  |  | | ✓ | |  |  |  |  |  |  |  |
| Tiong 2016 |  |  |  | |  | |  |  | ✓ |  |  |  |  |
| Tjam 2006 |  |  |  | | ✓ | |  |  |  |  |  |  |  |
| Toelle 2019 | ✓ |  |  | |  | |  |  |  |  |  |  |  |
| Torri 2018 |  |  |  | | ✓ | |  |  |  |  |  |  |  |
| Triantafyllidis 2018 | ✓ |  |  | |  | |  |  |  |  |  |  |  |
| Trinh 2018 |  |  |  | |  | |  |  |  |  | ✓ |  |  |
| Tucker 2019 |  | ✓ |  | |  | |  |  |  |  |  |  |  |
| Uhm 2017 | ✓ |  |  | |  | |  |  |  |  |  |  |  |
| Valle 2022 |  |  |  | | ✓ | |  |  |  |  |  |  |  |
| Valle 2022 |  |  | ✓ | |  | |  |  |  |  |  |  |  |
| VanBlarigan 2020 |  |  | ✓ | |  | |  |  |  |  |  |  |  |
| VanBlarigan 2020 | ✓ |  |  | |  | |  |  |  |  |  |  |  |
| VanVeen 2015 |  |  | ✓ | |  | |  |  |  |  |  |  |  |
| vanVeen 2015 |  |  |  | | ✓ | |  |  |  |  |  |  |  |
| Vandelanotte 2005 |  |  |  | |  | |  |  |  | ✓ |  |  |  |
| Vaughan 2021 |  |  |  | | ✓ | |  |  |  |  |  |  |  |
| Vloothuis 2018 |  |  |  | | ✓ | |  |  |  |  |  |  |  |
| Vluggen 2017 |  |  | ✓ | |  | |  |  |  |  |  |  |  |
| Vluggen 2018 |  | ✓ |  | |  | |  |  |  |  |  |  |  |
| Volders 2020 |  |  |  | | ✓ | |  |  |  |  |  |  |  |
| Volders 2020 | ✓ |  |  | |  | |  |  |  |  |  |  |  |
| vonStorch 2019 | ✓ |  |  | |  | |  |  |  |  |  |  |  |
| Vorrink 2017 | ✓ |  |  | |  | |  |  |  |  |  |  |  |
| Vorrink 2016 | ✓ |  |  | |  | |  |  |  |  |  |  |  |
| Walsh 2019 |  | ✓ |  | |  | |  |  |  |  |  |  |  |
| Wan 2020 |  |  |  | |  | |  |  |  |  |  |  | ✓ |
| Wang 2020 |  |  |  | |  | |  |  | ✓ |  |  |  |  |
| Wang 2017 |  |  | ✓ | |  | |  |  |  |  |  |  |  |
| Wang 2020 | ✓ |  |  | |  | |  |  |  |  |  |  |  |
| Wang 2018 |  |  | ✓ | |  | |  |  |  |  |  |  |  |
| Wangberg 2008 |  |  |  | |  | |  |  | ✓ |  |  |  |  |
| Webb 2019 | ✓ |  |  | |  | |  |  |  |  |  |  |  |
| Welch 2009 |  |  | ✓ | |  | |  |  |  |  |  |  |  |
| Whittemore 2019 |  |  | ✓ | |  | |  |  |  |  |  |  |  |
| Widmer 2014 |  |  | ✓ | |  | |  |  |  |  |  |  |  |
| Widmer 2016 |  | ✓ |  | |  | |  |  |  |  |  |  |  |
| Wienbarg 2018 | ✓ |  |  | |  | |  |  |  |  |  |  |  |
| Wiggers 2013 |  |  | ✓ | |  | |  |  |  |  |  |  |  |
| Wilczynska 2019 | ✓ |  |  | |  | |  |  |  |  |  |  |  |
| Willems 2013 |  |  | ✓ | |  | |  |  |  |  |  |  |  |
| Willems 2015 |  |  |  | |  | |  |  |  |  |  |  | ✓ |
| Winters-Stone 2018 |  | ✓ |  | |  | |  |  |  |  |  |  |  |
| Wong 2016 |  | ✓ |  | |  | |  |  |  |  |  |  |  |
| Wong 2021 | ✓ |  |  | |  | |  |  |  |  |  |  |  |
| Wright 2020 |  |  | ✓ | |  | |  |  |  |  |  |  |  |
| Wright 2012 |  | ✓ |  | |  | |  |  |  |  |  |  |  |
| Xu 2020 |  | ✓ |  | |  | |  |  |  |  |  |  |  |
| Yang 2019 | ✓ |  |  | |  | |  |  |  |  |  |  |  |
| Yin 2022 | ✓ |  |  | |  | |  |  |  |  |  |  |  |
| Zanaboni 2017 |  |  |  | | ✓ | |  |  |  |  |  |  |  |
| Zhang 2020 |  |  | ✓ | |  | |  |  |  |  |  |  |  |
| Zhang 2019 |  |  | ✓ | |  | |  |  |  |  |  |  |  |
| Zheng 2020 |  |  | ✓ | |  | |  |  |  |  |  |  |  |
|  |  |  |  | |  | |  |  |  |  |  |  |  |

1 Not Online/Web Based (n = 92)

2 Not a Randomised Control Trial (n = 80)

3 Conference abstracts (n = 64)

4 Intervention therapist led (n = 47)

5 Wrong outcome (n = 6)

6 Wrong comparison group (n = 2)

7 No PA or Diet component (n = 11)

8 Wrong patient population (n = 11)

9 Intervention focuses on monitoring (n = 6)

10 Unable to locate full text despite contacting author (n = 3)

11 Duplicate of another paper (n = 6)
